# Evaluating Strategies to Normalise Biological Replicates of Western Blot Data

**DOI:** 10.1371/journal.pone.0087293

**Published:** 2014-01-27

**Authors:** Andrea Degasperi, Marc R. Birtwistle, Natalia Volinsky, Jens Rauch, Walter Kolch, Boris N. Kholodenko

**Affiliations:** 1 Systems Biology Ireland, University College Dublin, Dublin, Republic of Ireland; 2 Department of Pharmacology and Systems Therapeutics, Icahn School of Medicine at Mount Sinai, New York, New York, United States of America; 3 Conway Institute of Biomolecular & Biomedical Research, University College Dublin, Dublin, Republic of Ireland; 4 School of Medicine and Medical Science, University College Dublin, Dublin, Republic of Ireland; Penn State College of Medicine, United States of America

## Abstract

Western blot data are widely used in quantitative applications such as statistical testing and mathematical modelling. To ensure accurate quantitation and comparability between experiments, Western blot replicates must be normalised, but it is unclear how the available methods affect statistical properties of the data. Here we evaluate three commonly used normalisation strategies: (i) by fixed normalisation point or control; (ii) by sum of all data points in a replicate; and (iii) by optimal alignment of the replicates. We consider how these different strategies affect the coefficient of variation (CV) and the results of hypothesis testing with the normalised data. Normalisation by fixed point tends to increase the mean CV of normalised data in a manner that naturally depends on the choice of the normalisation point. Thus, in the context of hypothesis testing, normalisation by fixed point reduces false positives and increases false negatives. Analysis of published experimental data shows that choosing normalisation points with low quantified intensities results in a high normalised data CV and should thus be avoided. Normalisation by sum or by optimal alignment redistributes the raw data uncertainty in a mean-dependent manner, reducing the CV of high intensity points and increasing the CV of low intensity points. This causes the effect of normalisations by sum or optimal alignment on hypothesis testing to depend on the mean of the data tested; for high intensity points, false positives are increased and false negatives are decreased, while for low intensity points, false positives are decreased and false negatives are increased. These results will aid users of Western blotting to choose a suitable normalisation strategy and also understand the implications of this normalisation for subsequent hypothesis testing.

## Introduction

Western blotting or protein immunoblotting, was introduced at the end of the 1970s to enable the detection of specific proteins [1], [2]. Although originally a qualitative or at best a semi-quantitative method, with the rise of computational systems biology [3], Western blotting has become increasingly important for fully quantitative applications. Two main applications are the parameterisation and validation of mathematical models of biological systems [4] and the testing of statistical significance between two or more experimental conditions or treatments [5].

Although technical aspects of Western blotting have improved over the years, for example by extending the linear range of detection [6], it is not yet clear how much quantitative information can be obtained and in which settings. Here we investigate the quantitative use of Western blotting, to determine its applicability and limits depending on the detection method and the data normalisation strategy used to quantitatively compare biological replicates of the same experimental conditions.

A requirement for the quantitative use of Western blot data is the linearity between quantified intensities and protein amounts. To detect and correct non-linearity, the authors in [7] suggest to use hyperbolic calibration curves to interpolate the correct relative concentration of the proteins of interest. These are dilution curves that need to be treated simultaneously to samples of interest, and in most situations constructing these dilution curves is not practical. Because this method is highly labour consuming and is not a laboratory common practice, we do not consider this approach in this paper. Nonetheless we investigate linearity in our Results section, where we quantify the extent of the linear range in the case of two detection systems: enhanced chemiluminescence (ECL) with X-ray film and ECL with charge coupled device (CCD) imager.

Although the topic of data normalisation has been widely explored in the context of microarrays [8], it has not yet been fully investigated in the context of Western blotting. For microarrays, such as single channel oligonucleotide arrays, the issue of data normalisation arises naturally when expression indices, obtained from gene probe sets intensities, need to be compared across different arrays, for example to identify differentially expressed genes [9]. In order to compare arrays quantitatively, several normalisation strategies have been proposed, where expression indices or intensities are scaled or transformed depending on the assumptions underlying each strategy. For example, assuming that the total amount of sample RNA is constant across arrays, the intensities are scaled such that the sum or the average of all intensities is equal across arrays (scaling methods [9]). Alternatively, assuming that the distribution of the intensities is conserved across arrays, the data is transformed such that the quantile-quantile plot of the intensities of the arrays approaches a straight line (quantile normalisation [9]). Or again, assuming that there is a set of genes whose expression index does not change across arrays, such as a set of housekeeping genes, this set can be used as reference (invariant set normalisation [10]).

**Figure 1:**
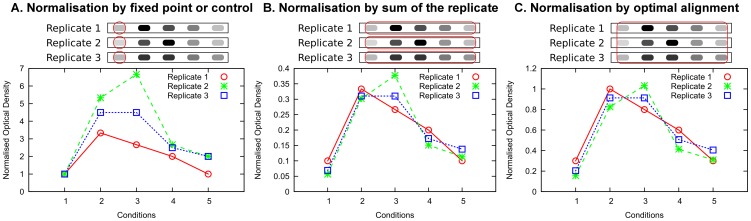
Normalisations of Western blot replicates in the literature. We divide the normalisations found in literature into three categories: (A) normalisation by fixed normalisation point or control; (B) normalisation by sum of the replicate; (C) normalisation by optimal alignment. For illustration purposes we do not use actual Western blot data. Each normalisation is presented using three cartoon Western blots, representing three replicates, and highlighting with red circles the data points used in the normalisation procedure. The graphs show the normalised data, where the points belonging to the same replicate are connected with lines.

In the case of Western blotting, usually a single protein is measured and a limited number of experimental conditions is on the same blot and is detected at the same time; a situation in stark contrast to microarrays where thousands of gene expression measurements are obtained for potentially many more conditions than is typically done by Western blotting. The data or measurements from a Western blot are obtained by dividing quantified intensities (optical densities – OD) by the intensities of appropriate reference proteins, e.g. housekeeping proteins, from the same samples. This procedure adjusts the intensities with respect to small variations in the number of cells and loading across samples within the same blot [11]–[13]. The need to normalise the data arises when comparing the results from biological replicates of the same experiment, for example to obtain statistical evidence that different conditions induce different protein amounts. We classify Western blot normalisations into three categories. The first and most widely used normalisation method is normalisation by fixed point (Figure 1A). It divides the data of a replicate by the measurement of a single condition, often referred to as control. It should be noted that although this shares similarities with the invariant set normalisation in the context of microarrays, the assumption that the reference condition is constant is not used and is in practice not satisfied. Thus the biological variability of the reference condition influences the variability of the normalised data. The second normalisation category we consider is normalisation by sum (Figure 1B), where the data on a blot is divided by the sum of the data on the same blot [14], or equivalently the data is scaled such that the average is the same across blots [15], [16]. It should be noted that in contrast with the analogous normalisation used in microarrays (scaling methods), in this case the sum is not assumed to be a constant. The biological variability of the sum and its dependency on the individual measurements might influence the variability of the normalised data. Most importantly, it is possible to compare two blots only if they present exactly the same conditions, using different lysates derived from cells cultured and treated in the same way. Yet, this condition is regularly met when producing a biological replicate. In our statistical formalisation we address the problem of characterising how the choice of reference (fixed point or sum) influences the normalised data. As third category we consider normalisation by optimal alignment (Figure 1C), where data from replicates are aligned using optimisation algorithms to minimise the uncertainty of the normalised data. Examples of this normalisation minimise either the sum of the squared differences between the replicates of each data point [17] or the coefficient of variation (CV) of the normalised data [18]. The assumption behind this method is that the measurements across replicates should preserve an overall trend.

To avoid the need for data normalisation, approaches for the absolute quantification of protein concentrations using Western blotting have been investigated [7], [19]. However, these methods are not widespread mainly due to increased experimental effort, in particular the need for purified proteins as standards. It is also possible to obtain replicates of lysates that are directly comparable by means of multi-strip Western blotting [20], where replicates are cut from different gels and blotted on the same membrane. However, multi-strip Western blots are typically used to compare more conditions on the same membrane, rather than replicates.

The quantitation of Western blots has also been the subject of theoretical investigations. In [21] the authors use a large amount of data to identify a suitable error model for Western blot data. Using the error model, they dissect the different sources of error, concluding that the main sources of variability are multiplicative and so log-normally distributed. Additionally, by removing the sources of error, they reduce the variability in the data significantly. This work is based on error models for microarray data [22], and is applicable only when a large amount of data is available. In [19], the authors suggest that technical errors can be reduced using a randomisation of time courses on a gel and smoothing the data using spline regression.

In this paper, first we discuss the problem of linearity between protein concentrations and quantified optical densities, which is a fundamental prerequisite to use Western blot data quantitatively in the absence of hard-to-obtain calibration curves. Second, we investigate how the choice of the normalisation strategy affects the normalised data. In particular, we evaluate the normalisations in terms of their ability to reduce variability in the data and of how they affect statistical decisions.

## Materials and Methods

### Sample Preparation

The MCF-7 cell line was maintained under standard conditions in Dulbecco's modified Eagle's medium supplemented with 10% foetal bovine serum. Cells were washed with ice cold phosphate buffered Saline and lysed in RIPA buffer (1% NP-40, 0.1% SDS, 0.5% Sodium deoxycholate, 50 mM Tris pH 7.5, 150 mM NaCl) supplemented with protease and phosphatase inhibitor cocktails (Sigma Aldrich) and protein concentration was quantitated by BCA protein assay (Invitrogen). Purified BSA (Applichem) was dissolved in RIPA buffer. Cell lysates and a BSA sample were serially diluted 1∶2 and run on SDS-PAGE using a standard protocol. Proteins were transferred to the PVDF (for ECL based detection) or Nitrocellulose (for LI-COR based proteins detection) membranes. Membranes were blocked with blocking solution (11500694001, Roche) for BSA detection or 5% skimmed milk for rest of the membranes. For Western blotting ERK (M-5670, Sigma Aldrich), mTOR (2972, Cell Signaling Technology), RSK1 (sc-231, Santa Cruz) and BSA (sc-50528, Santa Cruz) antibodies were used. Anti-rabbit HRP-conjugated (Cell Signaling Technology) or anti-Rabbit IR 800 (LI-COR) secondary antibodies were used for ECL or LI-COR protein detection systems, respectively. Signal was detected by standard X-ray films (Fuji), CCD camera (Advanced Molecular Vision) or LI-COR scanner.

### Image Acquisition and Densitometry

Several exposure times were tested for both ECL with film and ECL with CCD imager. In the case of the CCD imager we could choose the longest exposure that presented no signal saturation (overexposure), as detected by the software used in combination with the imager. In the case of X-ray film, we used our experience to select the films that had a good compromise between number of bands visible and the least possible exposure time. Films were digitalised using a high resolution CCD scanner (EPSON Perfection v750 Pro) without additional image corrections that could alter the linearity, such as automatic gain control [23]. Densitometry analysis was performed using the ImageJ Gel Analysis tool, where gel background was also removed individually for each band.

### Statistical Analysis of the Dilution Experiments

To assess the linearity of the dilution experiments we used linear regression and computed the coefficient of determination 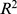 using Microsoft Excel software. Briefly, the closer the coefficient of determination is to 1, the more the linear model is appropriate to represent the data. For each detection method we tested different dilution ranges by determining 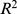 using the first *n* visible bands, i.e. starting from the least intense band that could be detected. For example, to test the linearity of the five dilutions range, reflecting a 32 fold difference, we computed the linear regression of the first six (*n* = 6) visible bands of a dilution curve and computed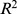. We then computed 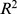 independently for three replicates and obtained mean and standard error. Using this approach we could compare coefficients of determination for specific dilution ranges across different detection methods. All quantified coefficients of determination of the dilution experiments can be found in Information S1.

### Theoretical Analysis of Normalisation Procedures

The theoretical analysis was performed developing dedicated scripts in the R language for statistical computing and implementing dedicated C++ programs. While R was used mainly to compute the results inferred from Western blot data, C++ programs were used to compute the results in the theoretical scenarios. To obtain samples from log-normal distributions, we computed samples from normal distributions using the Box-Muller method [24] and then exponentiated these samples with base *e*. Mean and variance of these normal distributions were calculated so that the mean and variance of the log-normal distributions were as desired. Combining samples from log-normal distributions we could obtain samples from the distributions of the normalised data, as defined in the text (Equations (2), (4) and (8)). Because the log-normal distributions were defined *a priori*, we could then estimate false positives and false negatives results by using t-tests as described in the corresponding figure legends. Briefly, a false positive is defined as a t-test result that yields a p-value lower than 0.05 when testing samples from two distributions that we defined as identical, while a false negative is defined as a t-test result that yields a p-value greater than 0.05 when testing samples from two distributions that we defined as different. Source files are available upon request from the corresponding author.

## Results

### Linearity between Protein Concentration and Quantified Optical Densities

**Figure 2:**
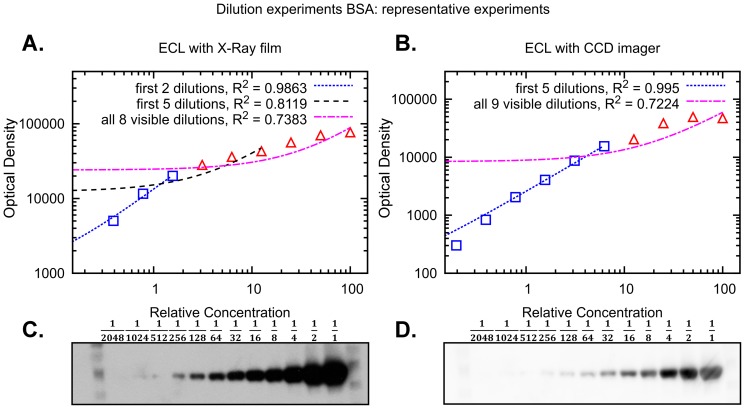
Signal linearity obtained by different Western blot detection systems. Representative experiments of Western blots containing 2-fold serial dilution of BSA. Shown are the representative results from 3 independent experiments. BSA was detected by (A,C) ECL with X-ray film and (B,D) ECL with CCD imager. Blue squares indicate data points that are linear, while red triangles indicate data points outside the linear range of detection. To highlight linear and non-linear data we use linear trend lines, reporting the coefficient of determination 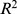. In (A,B) data are in log-log scale to improve visualisation.

In the absence of a calibration curve, a pre-requisite for obtaining quantitative Western blot data is a linear relationship between the amount of analyte and the measured intensity. To evaluate the extent of the linear range in commonly used detection methods, we prepared two 12 step 2-fold dilution series spanning a 2048-fold concentration range (three independent experiments). One sample series contained isolated Bovine Serum Albumin (BSA) while the other MCF-7 cell lysate. We used the first dilution series to quantify BSA and the second to quantify proteins across a mass range; they included Extracellular signal Regulated Kinases 1 and 2 (ERK1/2), ca. 40 kDa, Ribosomal protein S6 Kinase alpha-1 (RSK1), ca. 80 kDa, and Mammalian Target Of Rapamycin (mTOR), ca. 290 kDa. Proteins were detected using two detection systems: ECL with X-ray film and ECL with a CCD imager. Representative experiments with corresponding quantifications can be found in Figure 2 (BSA) and in Figures S1 (ERK), S2 (RSK1) and S3 (mTOR).

In order to identify the linear range of a dilution curve we used linear regression for an increasing number of data points, starting from the first detectable and least intense band of a curve. For each regression we then computed the coefficient of determination 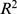, which indicates if the linear regression is a good model for the portion of the curve considered. The closer 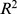 is to 1, the more linear the data is. After computing 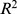 for each of three replicates we obtained mean and standard error.

As expected, our results show that both ECL with X-ray film and with CCD imager have a limited linear range. For example, the full dilution curve of BSA detected with ECL with CCD imager has an 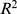 of 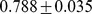.

Interestingly, we found that the linear range of ECL with CCD imager spans about five dilutions (32 fold). In particular, for all four proteins considered we obtained a significant reduction in 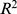 when we consider six dilutions (64 folds), with respect to five dilutions. For example, in the case of BSA, the coefficient of determination for five dilutions is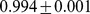, while for six dilutions it is reduced to 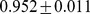, while for ERK the reduction is from 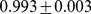 to 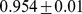.

ECL with X-ray film presented a smaller linear range than with CCD imager. In particular, for the five dilutions range where CCD imager is linear, X-ray film yields a sensibly lower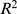. For example, for the BSA dilution this is reduced to 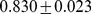, while for ERK to 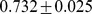. Linearity in the case of ECL with X-ray film seems to hold only for two or three dilutions, i.e. four or eight fold (Information S1).

**Figure 3:**
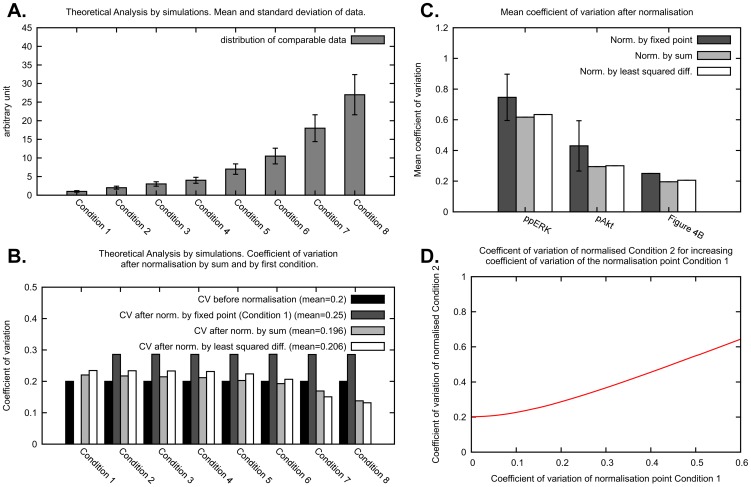
Effect of the normalisation on the CV of the normalised data. (A) Distribution of the data in a simulated scenario. In our theoretical analysis of the effects of the normalisation on the variability of the normalised data we consider a distribution of the response to eight conditions. We use log-normal distributions with CV 0.2 and mean of the response to the conditions from 1 to 8 as 1, 2, 3, 4, 7, 10.5, 18, 27. (B) CVs are shown for the distribution of the simulated data before normalisation, after normalisation by first condition, after normalisation by sum of all data points in a replicate and after normalisation by least squared differences. The mean CV is computed as the average across the eight conditions. (C) Data from Figure S3 of [25] (Figure S5 in this publication) were normalised using different normalisation strategies and the mean CV of the resulting normalised data is shown. As the mean CV obtained by the normalisation by fixed point depends on the choice of normalisation point, we report the mean and standard deviation obtained. We also report the mean CV obtained using ppERK and pAkt data and we compare them with the theoretical results of Figure 3B. (D) Before normalisation, the response to Condition 2 has a CV of 0.2, as shown in Figure 3A. Condition 2 is then normalised by fixed point, with Condition 1 as normalisation point. Here we show how the CV of normalised Condition 2 changes for increasing CV of the normalisation point Condition 1.

The difference between the two systems based on ECL is most likely due to saturation of the X-ray film by high intensity samples while trying to detect also the lowest intensity samples. This limitation can be avoided using a CCD imager, which uses a computerised image acquisition system, and is able to detect low intensity signals without high intensity signals becoming saturated as quickly as with film. Because we were able to avoid this overexposure, the non-linearity observed using the CCD imager (Fig. 3B) is likely due to antibody interactions, as suggested in [7].

Finally, we investigated the extent of the linear range when using secondary fluorescent antibodies of LI-COR to detect BSA and ERK (Figure S4). Results were comparable to what we described above for ECL with CCD imager.

In conclusion, the use of ECL with X-ray film for quantitative Western blotting should be limited to the case in which the intensities vary experimentally not more than 4 to 8 fold. ECL with CCD imager or secondary fluorescent antibody presents a wider linear range of about 32 fold.

### Formalisation of Normalisation Strategies for Western Blot Data

We now move the focus to the evaluation of the normalisation strategies that we categorised in the introduction. In this section we introduce a formalisation, i.e. a mathematical description, of the normalisation strategies we investigate. Without loss of generality, consider the measurements of a single target (e.g. a protein) under different conditions or treatments (e.g. inhibitors, stimuli) on the same blot. Each data point 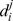, is indexed by the condition 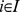
and the blot replicate number 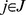. The standard experimental setups described above dictate the following:

1. Data points on one blot are comparable to one another, even if they come from different gel strips [20]. That is, 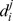 with different 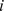 but with the same 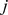 are directly comparable;

2. Data points on two different blots are not comparable. That is, 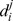 with different 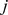 are not directly comparable. Normalisation must be employed to enable direct comparison across replicates.

Given the linearity conditions explored above are met and that the data points 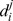 are samples from random variables 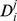, we have: 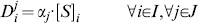
(1)where 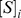 is the concentration of the protein of interest 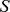 in condition 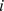, and 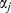 is the constant of proportionality of replicate 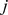. Note that if the replicates had been blotted on the same membrane using multi-strip Western blotting, all the data would be comparable with 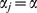 for all 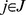.

The distribution of 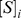 depends on the mean of the concentration of 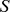 across a large number of cells in the lysate, on the biological variability, and on the technical error. We will now introduce the formalisation of the normalisation by fixed point or control, by sum of the replicate, and by optimal alignment of the replicates.

### Normalisation by a Fixed Normalisation Point or Control

In the normalisation by fixed normalisation point or control a band on the blot common to all replicates is chosen to be the normalisation point, and the data from a replicate are divided by the value of the normalisation point. In general, this normalisation can be applied choosing any band that is present on all replicates that need to be compared. The term “control” usually indicates the band of the untreated or neutral condition. Formally, this normalisation is a data transformation where the data points 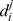 are substituted by the normalised data points 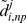 defined as: 
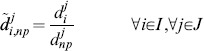
(2)


Where the index 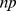 indicates a chosen normalisation point, which is an experimental condition all normalised data become relative to. In terms of random variables, the 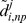 are samples from the random variables 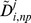 defined as: 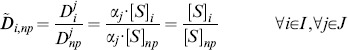
(3)


Notice that the random variable 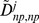 assumes value 1 with probability 1. Most importantly, the 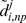 are now comparable across replicates 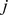, because the constants of proportionality 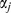 are all cancelled out. Additionally, all normalised data is dependent on the distribution of the normalisation point. In the following sections we will show how this dependency influences the variability of the normalised data and we will investigate how to choose a normalisation point.

### Normalisation by Sum of all Data Points in a Replicate

In the normalisation by sum, each data point on a replicate is divided by the sum of the values of all data points in that replicate. This way the data in each replicate becomes relative to this sum. It is important to ensure consistency of the sum across replicates, that is exactly the same conditions need to be part of the sum. This ensures that each data point is divided by a sample that comes from the same random variable. For example, in the presence of missing values, data points to be summed are chosen so that no replicate of the corresponding condition has a missing value. Without loss of generality, in this section we give a formalisation of normalisation by sum where we do not consider missing values.

In this normalisation, data points 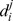 are divided by the sum of all data points in a replicate 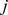. Formally, the normalised data 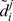 are defined as: 
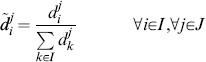
(4)


In terms of random variables, the normalised data points 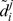 are samples from the random variables 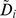 defined as: 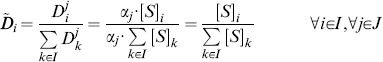
(5)


Similar to the normalisation by fixed point, the constants of proportionality 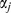 cancel and comparable normalised data are obtained. Notice that the normalised data are dependent on the value of all the data points in a replicate. The effects of this dependency on the variability of the data and hypothesis testing are investigated in the following sections. We note that Eq. 5 may be obtained by formulating the normalisation as an optimisation problem (see Information S2, Section S1).

### Normalisation by Optimal Alignment of the Replicates

In the normalisation by optimal alignment, the objective is to scale the data by a scaling factor for each replicate, so that replicates are aligned, that is the distance between data across replicates is minimal. This procedure has the specific goal of minimising the variability of the normalised data. Moreover, different notions of distance can be used, yielding different definitions of objective functions. The objective functions formalise the distance between the data in the replicates and are parametric with respect to the scaling factors. Finding the minimum of an objective function implies identifying optimal scaling factors. Examples of objective functions are as the sum of the squared differences between replicates [17] or the mean CV of the normalised data [18]. In the following we give a formal definition of normalisation by optimal alignment, considering a specific definition of distance, i.e. the sum of squared differences between replicates.

In this normalisation, each replicate 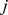 is scaled by a factor 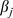 so that an optimal alignment of the replicates is achieved. It is necessary to avoid the trivial solution 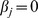 for all 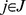, which can be done by introducing the constraint 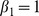 and estimating the remaining 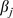. Here we consider the normalisation by least squared difference, defined as follows. Assuming we have 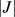 replicates and we want to align every replicate to the first replicate, the objective function for a least squared optimal alignment is as follows: 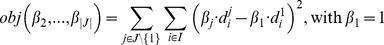



We minimise 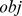 to find the optimal 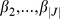. The result of this optimisation can be computed analytically and yields (see Information S2, Section S2 for the derivation): 
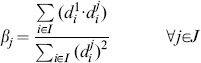
(7)


The normalised data 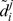 are defined as follows: 
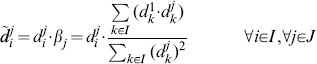
(8)


Notice that because of the definition of the 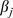, a normalised data point depends on a combination of the value of the data in the same replicate and the value of the data in replicate 1. More complex normalisations by optimal alignment of the replicates, such as the normalisation by minimisation of the mean CV of the normalised data in [18], may present normalised data points that are dependent on the values of all the data. For illustration purposes we use here normalisation by least squared difference as a representative of the normalisations by optimal alignment. We show in the Information S2, Equation (S5), that the data points normalised by least squared difference are all in the same unit, and are therefore directly comparable. In the next sections we investigate how the normalisations discussed above influence data variability and the statistical inference on data.

### Impact of Normalisation on Data Variability

A major aim of data normalisation is to make replicates suitable for quantitative comparison, while ensuring data integrity and avoiding adding uncertainty to the data. Here we show how different normalisation strategies affect the variability of the normalised data. We use the CV of the normalised data to compare the variability that results from applying the different normalisations.

For a theoretical investigation of how the choice of normalisation strategy affects the data, we use a simulated scenario. Suppose that the data of eight conditions or treatments is given as in Figure 3A. We chose a data distribution where the response to the treatments from one to eight has an increasing mean but the same CV of 0.2. In this and further analyses, we consider these distributions to be log-normal, because of the finding in [21] that the main sources of variability in Western blot data are multiplicative, and therefore log-normally distributed. In the Information S2, Section S3 and Figures S5 and S6, we replicate the results in this paper using normal distributions and obtain nearly identical results.

In Figure 3B we show how normalisation by fixed point, normalisation by sum and normalisation by least squared difference affect the CV of the eight conditions. To obtain these results we estimated the distributions associated with the random variables that we identified in Equation (3), Equation (5) and in the Information S2, Equation (S5), using a sampling approach based on the Box Muller sampling method [24]. We chose Condition 1 as the normalisation point for the normalisation by fixed point. It should be noted that because every condition is distributed with the same CV, this choice is an invariant in our analysis. The mean of the normalisation point does not determine the CV of the normalised data (data not shown), while we will show below that the CV of the normalised data depends strictly on the CV of the normalisation point chosen and that in practice data points with low mean, i.e. low OD, usually present higher CV. Normalisation by fixed point induced an increase in the mean CV, from 0.2 to 0.25, while increasing equally the CV of the response to each condition, with the obvious exception of Condition 1. Normalisation by sum slightly reduced the mean CV, from 0.2 to 0.196, while the effect on the single responses to the conditions is a redistribution of the CV in a way that is dependent on the mean of the conditions. Conditions with high mean present a reduced CV, while conditions with low mean present an increased CV (albeit slightly). This redistribution is due to the fact that the distribution of the data normalised by sum is dependent on the distribution of all the conditions, as can be seen in Equation (5). Normalisation by least squared difference optimisation increased very slightly the mean CV, from 0.2 to 0.206, while the effect on the single responses to the conditions is a redistribution of the CV analogous to what is observed for the normalisation by sum.

In order to obtain evidence that support the above theoretical investigation, we apply the normalisations to our Western blot data published in Figure S3 of [25] and also available in Figure S7. These data are composed of two data sets, one with measurements of phosphorylated ERK (ppERK), and the other with measurements of phosphorylated Akt (pAkt). Each data set is composed of three replicates of multi-strip Western blots, where on each blot there are 70 conditions divided into seven time courses for two different cell lines. Each data set is thus composed of 210 data points of measurements of band intensities already divided by the intensity of the corresponding loading controls, which are total ERK and Akt, respectively. These blots were done using ECL for protein detection and CCD imaging for recording of band intensities. All measurements were detected avoiding overexposure, and as most of the measurements are within a limited dynamic range they are likely within a linear range of detection. Figure 3C illustrates the mean CV obtained for the two data sets after applying different normalisation strategies, and compares these results with the theoretical investigation of Figure 3B. The results obtained with the experimental data agree with the theoretical investigation. The mean CV of the normalised data is relatively low for the normalisation by sum and the normalisation by least squared difference, while higher, on average, for the normalisation by fixed point. In practice, the result of the normalisation by fixed point depends on the choice of the normalisation point, yielding normalised data with low and high variability depending on such choice. In the next section we investigate how to choose a normalisation point.

### Low Intensity Data Points are Unsuitable Normalisation Points

In this section we investigate how, in the normalisation by fixed point, the choice of the normalisation point affects the variability of the data. In Figure 3D we illustrate how an increase in the CV of the normalisation point (Condition 1) induces a monotonic increase in the CV of the distribution of the normalised data (Condition 2), estimated using Equation (3). This result implies that the CV of the data normalised by fixed point is directly correlated with the CV of the particular condition used as normalisation point. This result also implies that a good choice for a normalisation point is a condition that presents a response with low CV, and hence low uncertainty. Although for non-comparable biological replicates it is impossible to pinpoint which data points have low variability, in the following we provide evidence that low protein band intensities usually yield normalised data with high variability.

**Figure 4:**
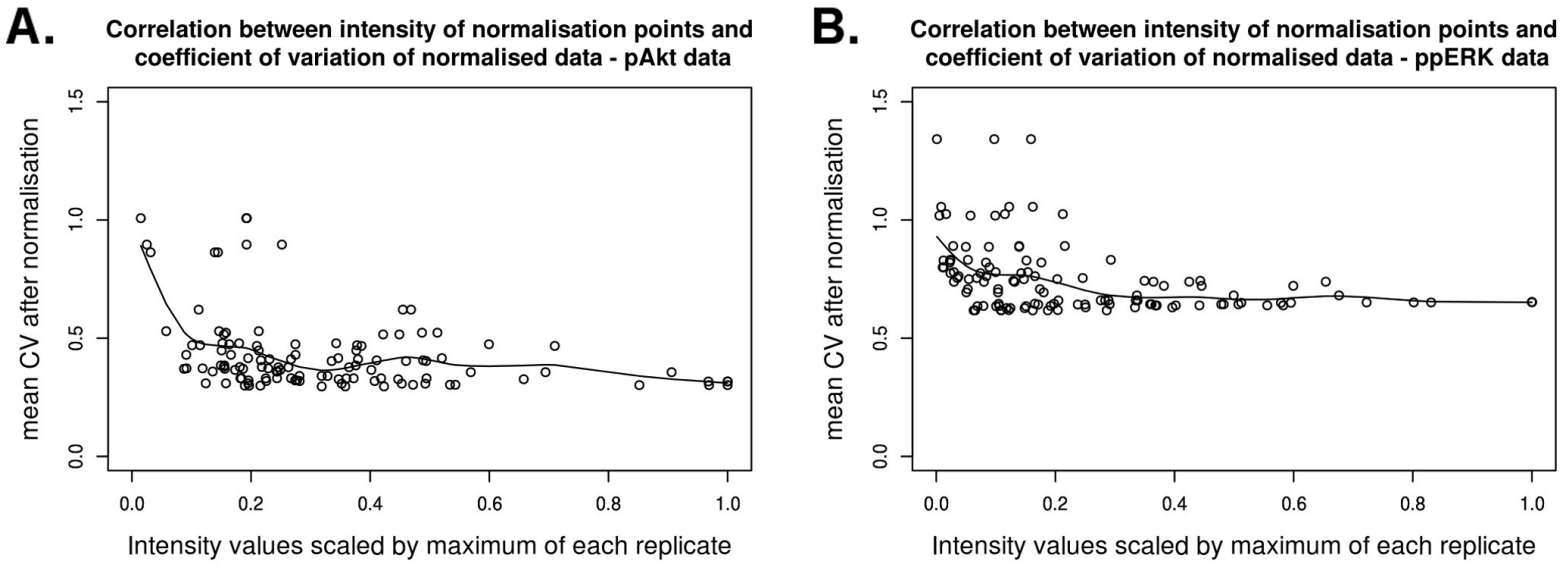
Correlation between the intensity of the normalisation points and the CV of the normalised data. Using data from (A) phosphorylated Akt and (B) phosphorylated ERK from Figure S3 in [25] (Figure S5 in this publication) we tested every point on a blot as normalisation point. For each resulting normalisation we computed the average of the CV of the normalised data points, and plotted the value of each data point (scaled so that the maximum of each replicate is equal to 1) against the average CV obtained by normalising with the corresponding data point. The result shows how the intensities of each normalisation point chosen correlate with the variability of the normalised data.

For this analysis we again use the Western blot data in Figure S7. For both ppERK and pAkt data sets we use each data point as normalisation point and calculate the average CV of the other points resulting from this normalisation. The results are illustrated in Figure 4 where, using a regression by spline functions, we show that choosing low intensity bands as a normalisation point causes an increase in the mean CV of the normalised data. Additionally, because of the result in Figure 3D, we can infer that low intensity bands have usually a larger CV and thus a higher uncertainty. This is most likely due to the low signal-to-noise ratio or, in other words, due to the presence of background noise and the difficulty to separate this noise from low intensity measurements.

In addition, we investigated whether the presence of data points that are outside the linear range of detection could also affect the choice of normalisation point. We performed an analysis analogous to what we described above for Figure 4 using the data from the three replicates of the dilution experiments illustrated in Figure 2 and Figures S1, S2 and S3. We used the data of the ECL with CCD imager detection system, which all present non-linearity outside the 32 fold linear range. The results for proteins BSA, RSK1 and mTOR present similarities with what we have found for ppERK and pAkt in Figure 4, i.e. using lower intensity measurements as normalisation point induces larger CV. The result for ERK is shown in Figure S8 and indicates that in this case both high intensity and low intensity normalisation points induce high CV, while medium intensity measurements induce the smallest CV. This is most likely due to the fact that the hyperbolic part of the dilution curves, which is composed of high intensity bands, is not reproduced consistently across replicates. Thus, the variability of high intensity data that are outside the linear range can induce normalised data with large CV even when a high intensity measurement is used as normalisation point.

### Impact of Normalisation on Statistical Testing

In this section we use a simulated scenario to investigate the effects of normalisation on the statistical testing applied to examine the significance of differences between protein bands detected by Western blotting. In particular, we test how normalisations influence the sensitivity and specificity of the two-tailed t-test [5], which is frequently used. In order to evaluate the sensitivity and the specificity, we estimate the percentage of false positives and false negatives by repeated data sampling.

It is standard practice to employ the t-test in spite of the fact that the actual distribution of Western blot data is unknown and theoretical investigation points toward a log-normal distribution [21], which is different from the normal distribution assumed in the test. Fortunately, the t-test is robust with respect to this violation of its assumptions [26], [27] having enabled its widespread application to Western blot data. As we want our analysis to be relevant for the practitioners we therefore comply with the established practice, and also use the t-test.

**Figure 5:**
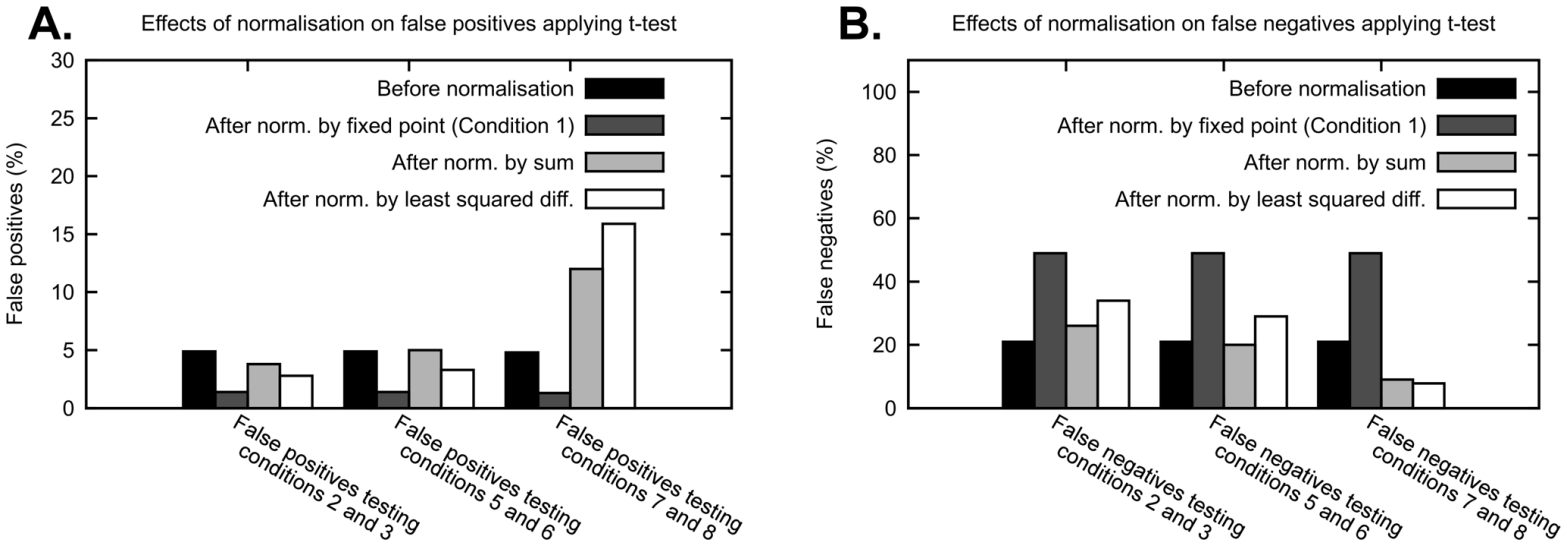
Effects of normalisation on false positives and false negatives when applying t-test for equality of the mean. (A) We consider responses to eight conditions with log-normal distributions with CV of 0.2 and means of the conditions from 1 to 8 equal to: 1, 2, 2, 4, 7, 7, 18, 18. A number n = 5 of sampled replicates are obtained from these distributions and normalised using the normalisations above. Using these replicates before and after normalisation, conditions are tested using a two-tailed t-test with threshold p-value of 0.05. We repeat this procedure a large number of times and estimate the percentage of false positives. (B) In analogy with (A), we estimate the number of false negatives considering means of the conditions from 1 to 8 equal to: 1, 2, 3, 4, 7, 10.5, 18, 27. Notice that for a fair comparison, when testing two conditions, one has a mean that is always 2/3 the mean of the other, e.g. Condition 5 has mean 7 and Condition 6 has mean 10.5, with 7/10.5 = 2/3.

In Figures 5A,B, the row labelled “Before normalisation” illustrates the expected percentage of false positives and false negatives obtained applying the t-test to data distributed as described in the previous figures. Because we set a threshold p-value of 0.05 in the t-test, the percentage of false positive is about 5%, as expected. Variations to the percentage of false positives and false negatives should be attributed to the application of the normalisations.

Normalisation by fixed point reduces the percentage of false positives, but greatly increases the percentage of false negatives, i.e. the specificity of the test is increased but the sensitivity is greatly reduced. This result is in agreement with our finding that the normalisation by fixed point increases the CV of the data. Thus, choosing this normalisation method will fail to identify some of the differences between data points. Normalisation by sum affects the percentage of false positives and false negatives in a way that is dependent on the mean of the response to the conditions tested. If relatively low values are tested, e.g. Conditions 2 and 3 in Figures 5A,B, the number of false positives decreases and the number of false negatives increases, while if relatively high values are tested, e.g. Conditions 7 and 8, the number of false positives increases and the number of false negatives decreases. Normalisation by least squared difference also affects false positives and false negatives depending on the magnitude of the data tested. Additionally, it seems that the normalisations by optimal alignment, such as by least squared difference, induce a stronger change in the sensitivity and specificity than the normalisation by sum. In general, normalisation by sum and by optimal alignment can introduce false positives when testing data with values relatively higher than the rest of the data set, reducing the specificity of the test.

## Discussion

In this paper we have investigated two issues that are important for the quantitative use of Western blot data, i.e. linearity of the detection system and the influence of data normalisations. Our results indicate that for quantitative Western blotting, if the measured intensities vary more than 4–8 fold, then the ECL detected by CCD imager system is preferable to ECL detected by X-ray film, as it yields a larger linear dynamic range. The linear range in the case of ECL in combination with CCD imager spans about 32 fold concentration change for four different proteins with different molecular mass, (Figure 2 and Figures S1, S2 and S3). When we tested fluorescent secondary antibodies detected by the LI-COR scanner, we found that a linear range is similar to the ECL with CCD imager detection method (Figure S4).

To better understand the mechanisms behind the normalisation of Western blot data, we use a formalisation based on statistical arguments of three normalisation strategies. Our findings reveal that the normalisation by fixed point introduces additional variability in the data (Figure 3B), and that conditions that induce responses with low CV are preferable normalisation points, because they induce a lower CV of the normalised data (Figure 3D). Although the CV of the response to specific conditions is in general not known, we provide evidence of whether low, medium or high intensity measurements have usually high or low CV. In particular we showed that low intensity measurements are usually inappropriate normalisation points (Figure 4). This is most likely due to the low signal-to-noise ratio and consequent high CV of low intensity measurements. Additionally, we showed that high intensity measurements that are outside the linear dynamic range are inappropriate normalisation points (Figure S8). Therefore, we suggest that for this type of normalisation the most appropriate normalisation points are data points with medium intensity measurements. Because the normalisation by fixed point increases the CV of the normalised data, this also has an impact on statistical testing. When applying a two-tailed t-test to the normalised data, we saw an increase in the specificity of the test and a strong decrease of the sensitivity (Figure 5A,B). While a high specificity is desirable, the decline in sensitivity increases the chances of overlooking significant differences between data points. In addition, if the normalisation point is not chosen carefully, the normalised data could present a high variability and it might become very difficult to detect when two conditions yield different results.

The normalisations by sum and by optimal alignment also influence the variability of the normalised data. Rather than introducing uncertainty, in this case the uncertainty is redistributed depending on the relative magnitude of the measurements (Figures 3A and 3B). In particular, the variability of high intensity measurements is reduced, while the variability of low intensity measurements is increased. This redistribution is due to the fact that normalised data points depend on the data points from other conditions or even from other replicates, as highlighted by the random variable of the normalised data in Equation (5) and Equation (S5) in Information S2.

A consequence of this redistribution is also that normalisations by sum and by optimal alignment have an impact on statistical testing. By applying a two-tailed t-test we observed an increase in sensitivity and decrease in specificity, when testing conditions with high intensity measurements (Figures 5A,B). Because more false positives are detected, the distinction of differences between two data points with high intensity measurements becomes less reliable than before normalisation. The alterations of sensitivity and specificity are inverted when data points with low intensity measurements are tested. These results imply that when these normalisations are applied, it is necessary to pay attention to whether high intensity or low intensity data points are tested and interpret the results accordingly. It is also possible to envision the definition of a data transformation or modified t-test to tune sensitivity and specificity based on the relative magnitude of the measurements tested, and calibrate the number of false positives to 5% of the cases.

Our findings also have implications for the use of Western blot data for mathematical model training and validation. In this setting, data is compared to the output of a model and appropriate values for the parameters of the model are identified, aiming to obtain the best possible agreement between data and output [4]. Because data normalisation has an influence on the distribution of the normalised data, we advise to normalise also the model output before comparing it to the data, when the nature of the mathematical model permits it. This should allow for a fair comparison between output and data, because in principle they would be subject to the same data transformation.

Although the quantitative use of Western blotting is now widespread, published articles often lack the details of how Western blot results were quantified and how biological replicates were compared to obtain statistics [23]. We hope that the results in this paper will serve as a reference and encourage scientists to include in future publications what we demonstrate to be critical information. To this end, based on our results, we wrote a short manual of one page that contains a step-by-step guide to help biologists choose the normalisation strategy that is the most appropriate to their case. This manual can be found in Information S3.

## Supporting Information

Figure S1
**Signal linearity of ERK obtained by different Western blot detection systems.** Shown are representative results from three independent experiments of Western blots containing 2-fold serial dilution of cell lysate. ERK was detected by (A,C) ECL with X-ray film and (B,D) ECL with CCD imager. Blue squares indicate data points that are linear, while red triangles indicate data points outside the linear range of detection. To highlight linear and non-linear data we use linear trend lines, reporting the coefficient of determination 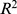. In (A,B) data are in log-log scale to improve visualisation.(TIFF)

Figure S2
**Signal linearity of RSK1 obtained by different Western blot detection systems.** Shown are representative results from three independent experiments of Western blots containing 2-fold serial dilution of cell lysate. RSK1 was detected by (A,C) ECL with X-ray film and (B,D) ECL with CCD imager. Blue squares indicate data points that are linear, while red triangles indicate data points outside the linear range of detection. To highlight linear and non-linear data we use linear trend lines, reporting the coefficient of determination 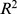. In (A,B) data are in log-log scale to improve visualisation.(TIFF)

Figure S3
**Signal linearity of mTOR1 obtained by different Western blot detection systems.** Shown are representative results from three independent experiments of Western blots containing 2-fold serial dilution of cell lysate. Protein mTOR1 was detected by (A,C) ECL with X-ray film and (B,D) ECL with CCD imager. Blue squares indicate data points that are linear, while red triangles indicate data points outside the linear range of detection. To highlight linear and non-linear data we use linear trend lines, reporting the coefficient of determination 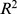. In (A,B) data are in log-log scale to improve visualisation.(TIFF)

Figure S4
**Signal linearity of BSA and ERK obtained by fluorescent secondary antibodies.** Shown are representative results from three independent experiments of Western blots containing 2-fold serial dilution of (A,C) BSA and (B,D) cell lysate. BSA and ERK were detected using fluorescent secondary antibodies. Blue squares indicate data points that are linear, while red triangles indicate data points outside the linear range of detection. To highlight linear and non-linear data we use linear trend lines, reporting the coefficient of determination 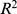. In (A,B) data are in log-log scale to improve visualisation.(TIFF)

Figure S5 **Effect of the normalisation on the coefficient of variation of the normalised data.** (A) CVs are shown for the distribution of the simulated data before normalisation, after normalisation by first condition, after normalisation by sum of all data points in a replicate and after normalisation by least squared differences. The mean coefficient of variation is computed as the average across the eight conditions. Mean and standard deviation of the data before normalisation is given in Figure 3A of the main text, and here is normally distributed. (B) Before normalisation, the response to Condition 2 has a coefficient of variation of 0.2, as shown in Figure 3A of the main text. Condition 2 is then normalised by fixed point, with Condition 1 as normalisation point. Here we show how the coefficient of variation of normalised Condition 2 changes for increasing coefficient of variation of the normalisation point Condition 1.
(TIFF)

Figure S6 **Effects of normalisation on false positives and false negatives when applying t-test for equality of the mean.** (A) We consider responses to eight conditions with normal distributions with CV of 0.2 and means of the conditions from 1 to 8 equal to: 1, 2, 2, 4, 7, 7, 18, 18. A number n = 5 of sampled replicates are obtained from these distributions and normalised using the normalisations above. Using these replicates before and after normalisation, conditions are tested using a two-tailed t-test with threshold p-value of 0.05. We repeat this procedure a large number of times and estimate the percentage of false positives. (B) In analogy with (A), we estimate the number of false negatives considering means of the conditions from 1 to 8 equal to: 1, 2, 3, 4, 7, 10.5, 18, 27. Notice that for a fair comparison, when testing two conditions, one has a mean that is always 2/3 the mean of the other, e.g. Condition 5 has mean 7 and Condition 6 has mean 10.5, with 7/10.5 = 2/3.
(TIFF)

Figure S7 **Figure S3 of** [**25**] **.** Experimental data used in Figures 3C and 4. The experiments shown in Figure S5 were performed as described by Rauch et al. in [25].
(TIFF)

Figure S8 **Correlation between the intensity of the normalisation points and the CV of the normalised data.** Using data from the three replicates of the ERK dilution experiments detected with CCD imager, we tested every point on a blot as normalisation point. For each resulting normalisation we computed the average of the CV of the normalised data points, and plotted the value of each data point (scaled so that the maximum of each replicate is equal to 1) against the average CV obtained by normalising with the corresponding data point. The result shows how the intensities of each normalisation point chosen correlate with the variability of the normalised data.
(TIFF)

Information S1 **Data and statistical analysis of dilution experiments for BSA, ERK, RSK1 and mTOR.**
(PDF)

Information S2 **Mathematical Supplement.** This document contains a characterisation of the normalisation by sum as an optimisation problem, an analytical solution of the normalisation by least squared difference optimisation and a description of how the results in Figures S5 and S6 were obtained using normal distributions.
(DOC)

Information S3 **Guide lines for the quantification of Western blots.**
(DOC)
