# Supplementary figures and images for: Evaluating Strategies to Normalise Biological Replicates of Western Blot Data
Source: PLoS One. 2014 Jan 27;9(1):e87293. doi: 10.1371/journal.pone.0087293 (PMC3903630; doi:10.1371/journal.pone.0087293)

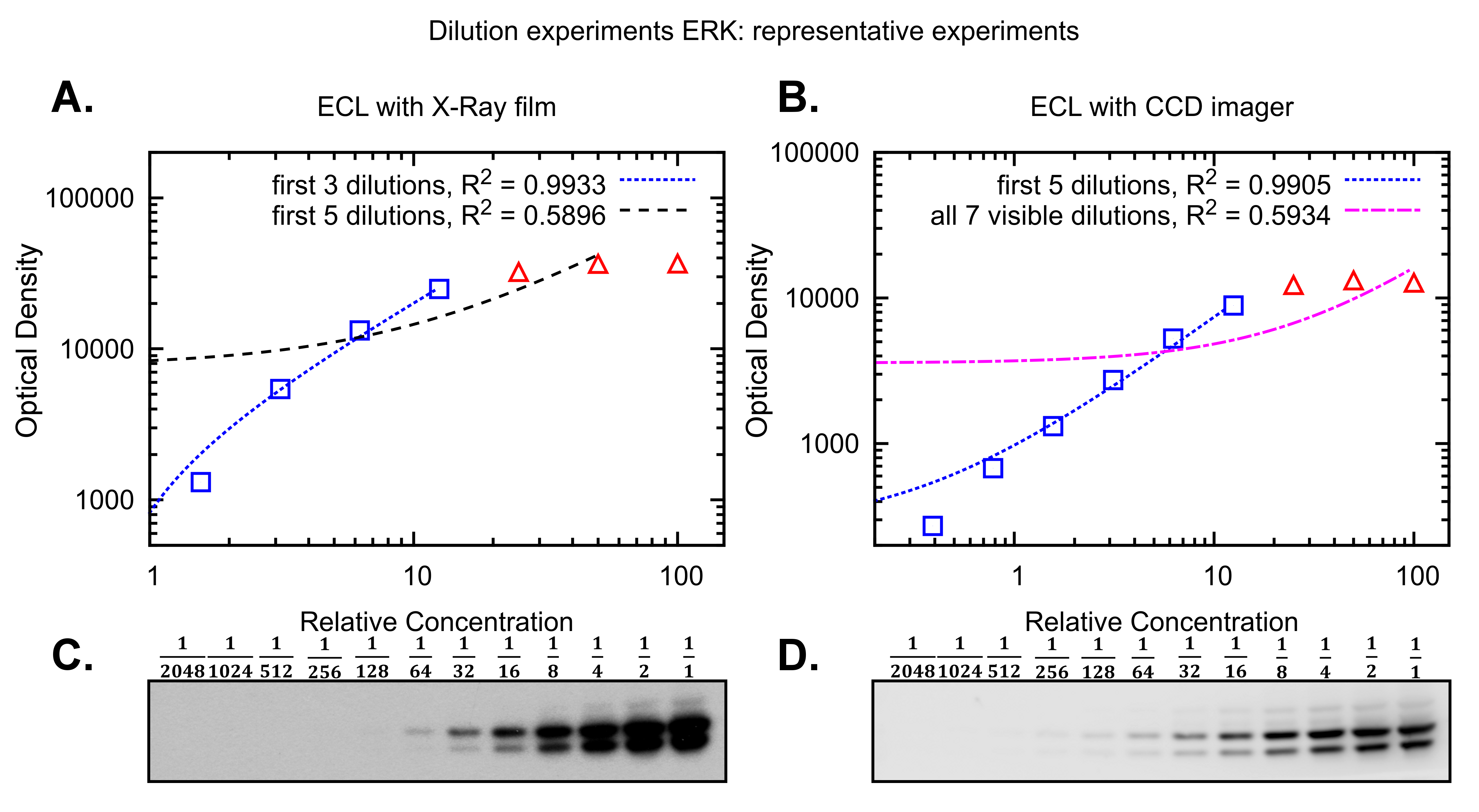

Supplement: Figure S1 — Signal linearity of ERK obtained by different Western blot detection systems. Shown are representative results from three independent experiments of Western blots containing 2-fold serial dilution of cell lysate. ERK was detected by (A,C) ECL with X-ray film and (B,D) ECL with CCD imager. Blue squares indicate data points that are linear, while red triangles indicate data points outside the linear range of detection. To highlight linear and non-linear data we use linear trend lines, reporting the coefficient of determination . In (A,B) data are in log-log scale to improve visualisation. (TIFF) [file pone.0087293.s001.tiff]

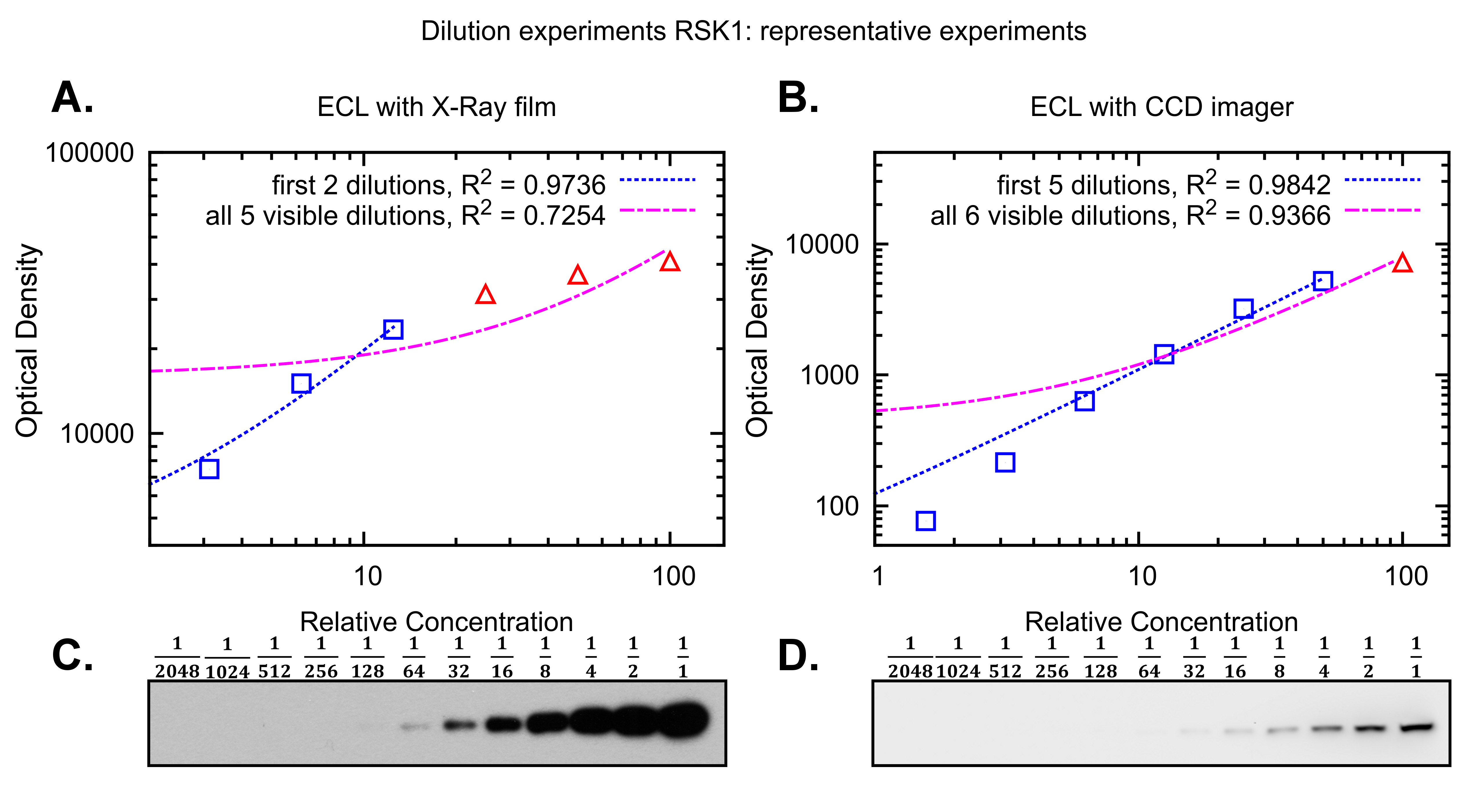

Supplement: Figure S2 — Signal linearity of RSK1 obtained by different Western blot detection systems. Shown are representative results from three independent experiments of Western blots containing 2-fold serial dilution of cell lysate. RSK1 was detected by (A,C) ECL with X-ray film and (B,D) ECL with CCD imager. Blue squares indicate data points that are linear, while red triangles indicate data points outside the linear range of detection. To highlight linear and non-linear data we use linear trend lines, reporting the coefficient of determination . In (A,B) data are in log-log scale to improve visualisation. (TIFF) [file pone.0087293.s002.tiff]

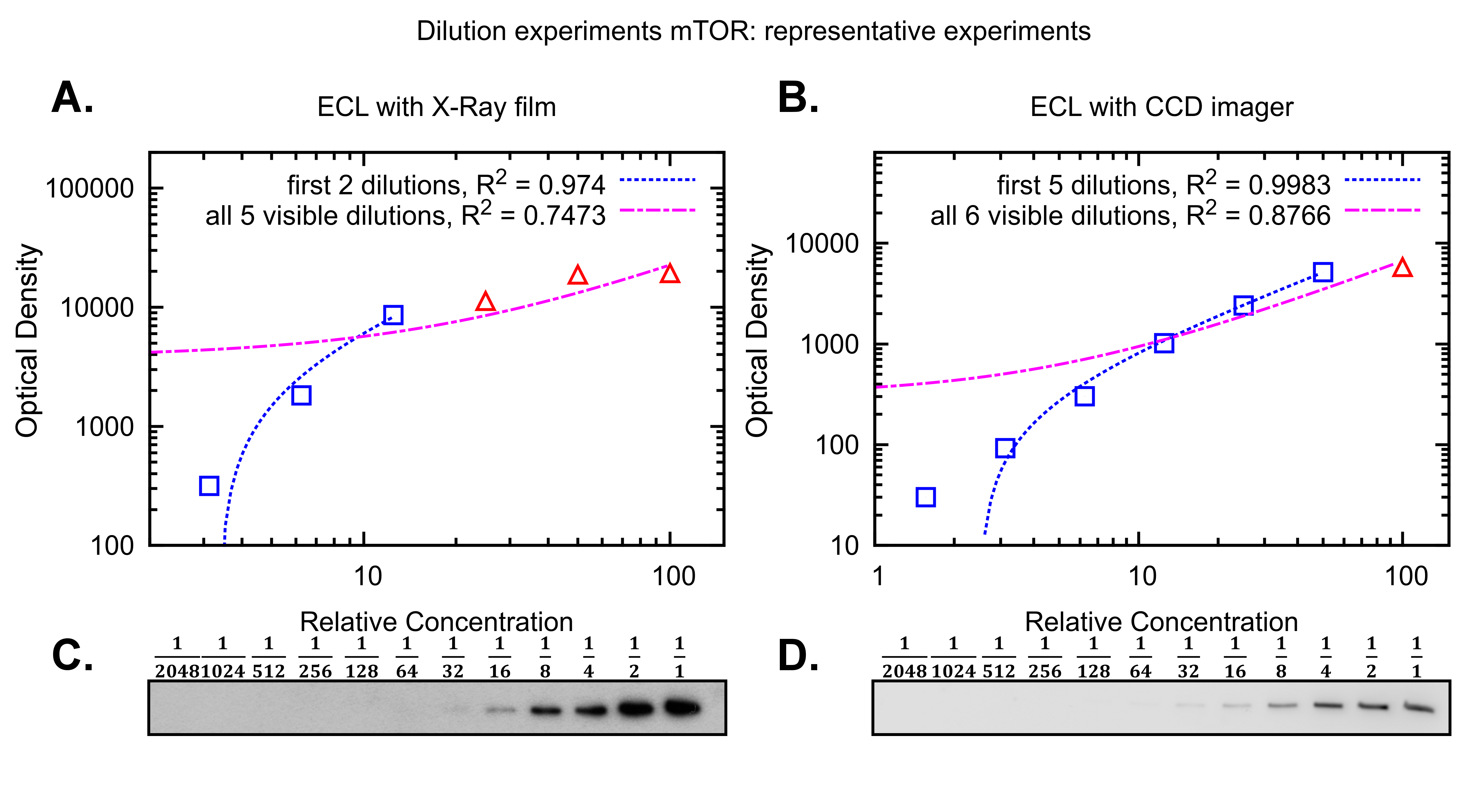

Supplement: Figure S3 — Signal linearity of mTOR1 obtained by different Western blot detection systems. Shown are representative results from three independent experiments of Western blots containing 2-fold serial dilution of cell lysate. Protein mTOR1 was detected by (A,C) ECL with X-ray film and (B,D) ECL with CCD imager. Blue squares indicate data points that are linear, while red triangles indicate data points outside the linear range of detection. To highlight linear and non-linear data we use linear trend lines, reporting the coefficient of determination . In (A,B) data are in log-log scale to improve visualisation. (TIFF) [file pone.0087293.s003.tiff]

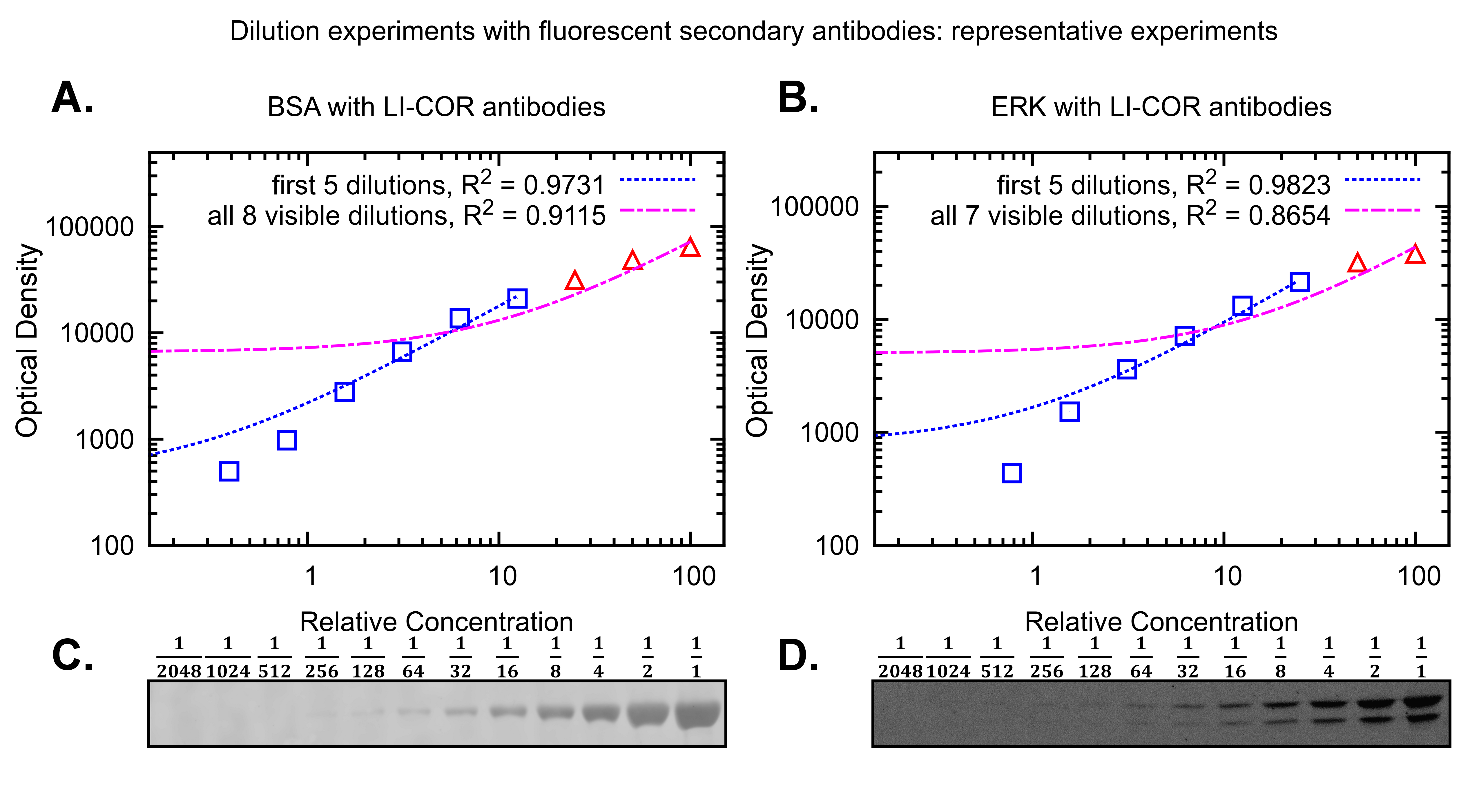

Supplement: Figure S4 — Signal linearity of BSA and ERK obtained by fluorescent secondary antibodies. Shown are representative results from three independent experiments of Western blots containing 2-fold serial dilution of (A,C) BSA and (B,D) cell lysate. BSA and ERK were detected using fluorescent secondary antibodies. Blue squares indicate data points that are linear, while red triangles indicate data points outside the linear range of detection. To highlight linear and non-linear data we use linear trend lines, reporting the coefficient of determination . In (A,B) data are in log-log scale to improve visualisation. (TIFF) [file pone.0087293.s004.tiff]

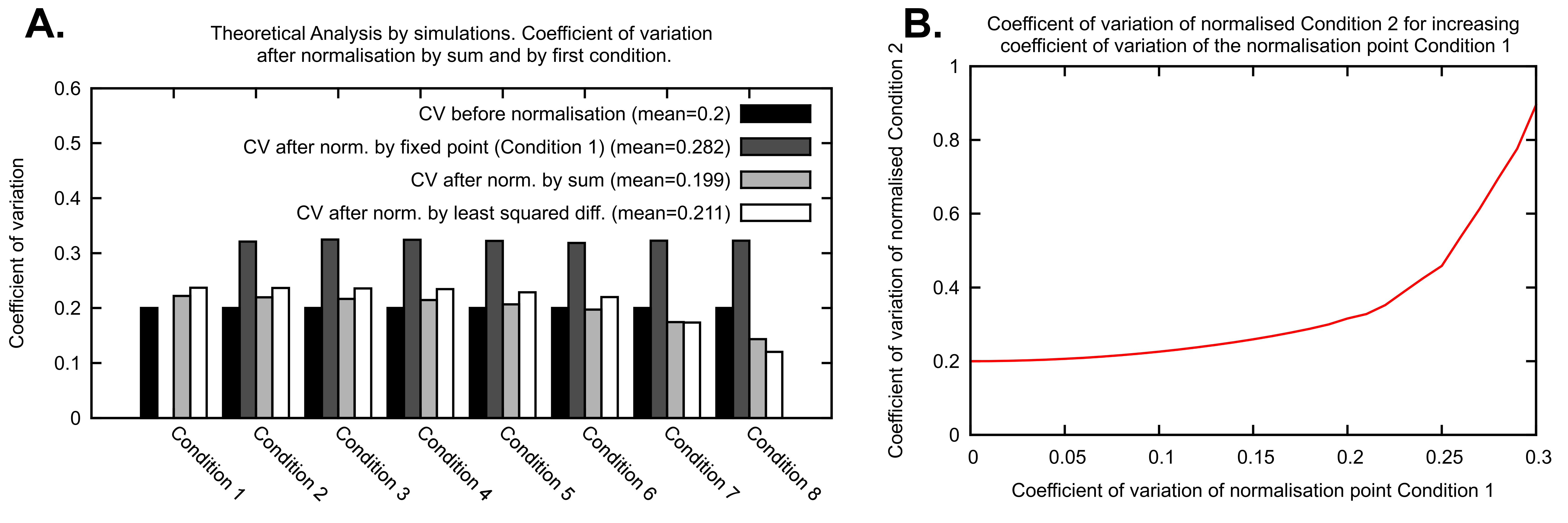

Supplement: Figure S5 — Effect of the normalisation on the coefficient of variation of the normalised data. (A) CVs are shown for the distribution of the simulated data before normalisation, after normalisation by first condition, after normalisation by sum of all data points in a replicate and after normalisation by least squared differences. The mean coefficient of variation is computed as the average across the eight conditions. Mean and standard deviation of the data before normalisation is given in Figure 3A of the main text, and here is normally distributed. (B) Before normalisation, the response to Condition 2 has a coefficient of variation of 0.2, as shown in Figure 3A of the main text. Condition 2 is then normalised by fixed point, with Condition 1 as normalisation point. Here we show how the coefficient of variation of normalised Condition 2 changes for increasing coefficient of variation of the normalisation point Condition 1. (TIFF) [file pone.0087293.s005.tiff]

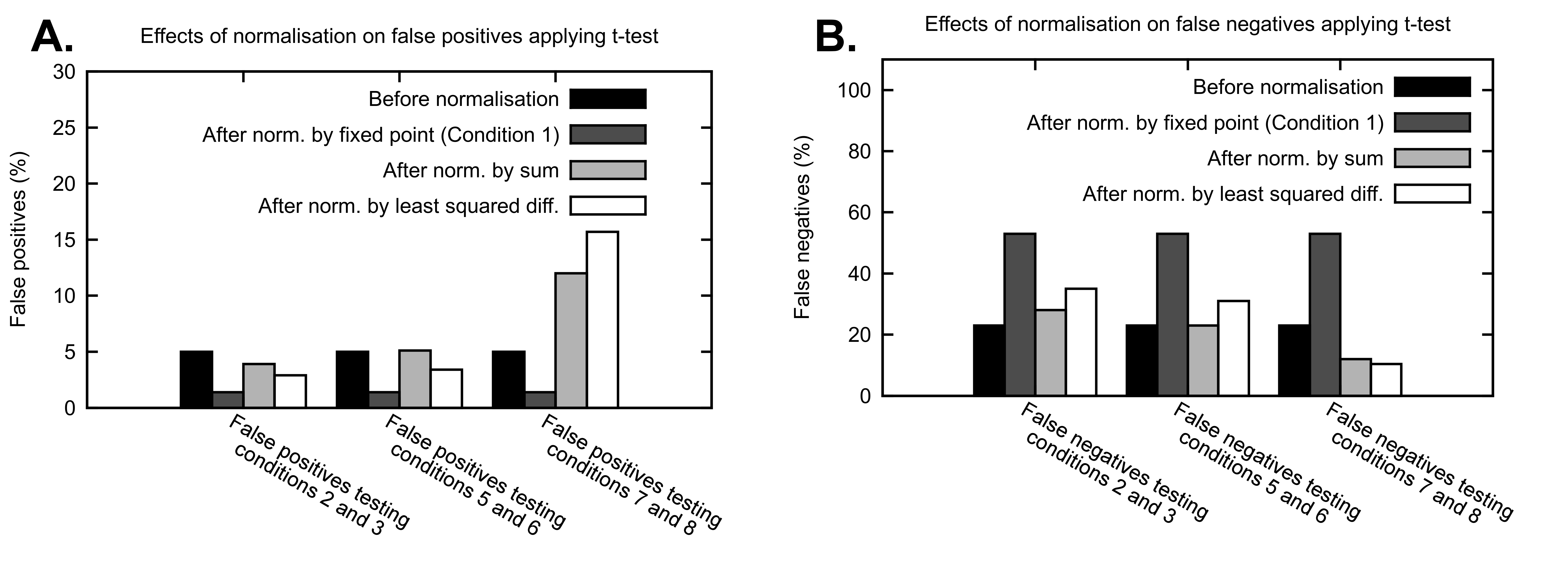

Supplement: Figure S6 — Effects of normalisation on false positives and false negatives when applying t-test for equality of the mean. (A) We consider responses to eight conditions with normal distributions with CV of 0.2 and means of the conditions from 1 to 8 equal to: 1, 2, 2, 4, 7, 7, 18, 18. A number n = 5 of sampled replicates are obtained from these distributions and normalised using the normalisations above. Using these replicates before and after normalisation, conditions are tested using a two-tailed t-test with threshold p-value of 0.05. We repeat this procedure a large number of times and estimate the percentage of false positives. (B) In analogy with (A), we estimate the number of false negatives considering means of the conditions from 1 to 8 equal to: 1, 2, 3, 4, 7, 10.5, 18, 27. Notice that for a fair comparison, when testing two conditions, one has a mean that is always 2/3 the mean of the other, e.g. Condition 5 has mean 7 and Condition 6 has mean 10.5, with 7/10.5 = 2/3. (TIFF) [file pone.0087293.s006.tiff]

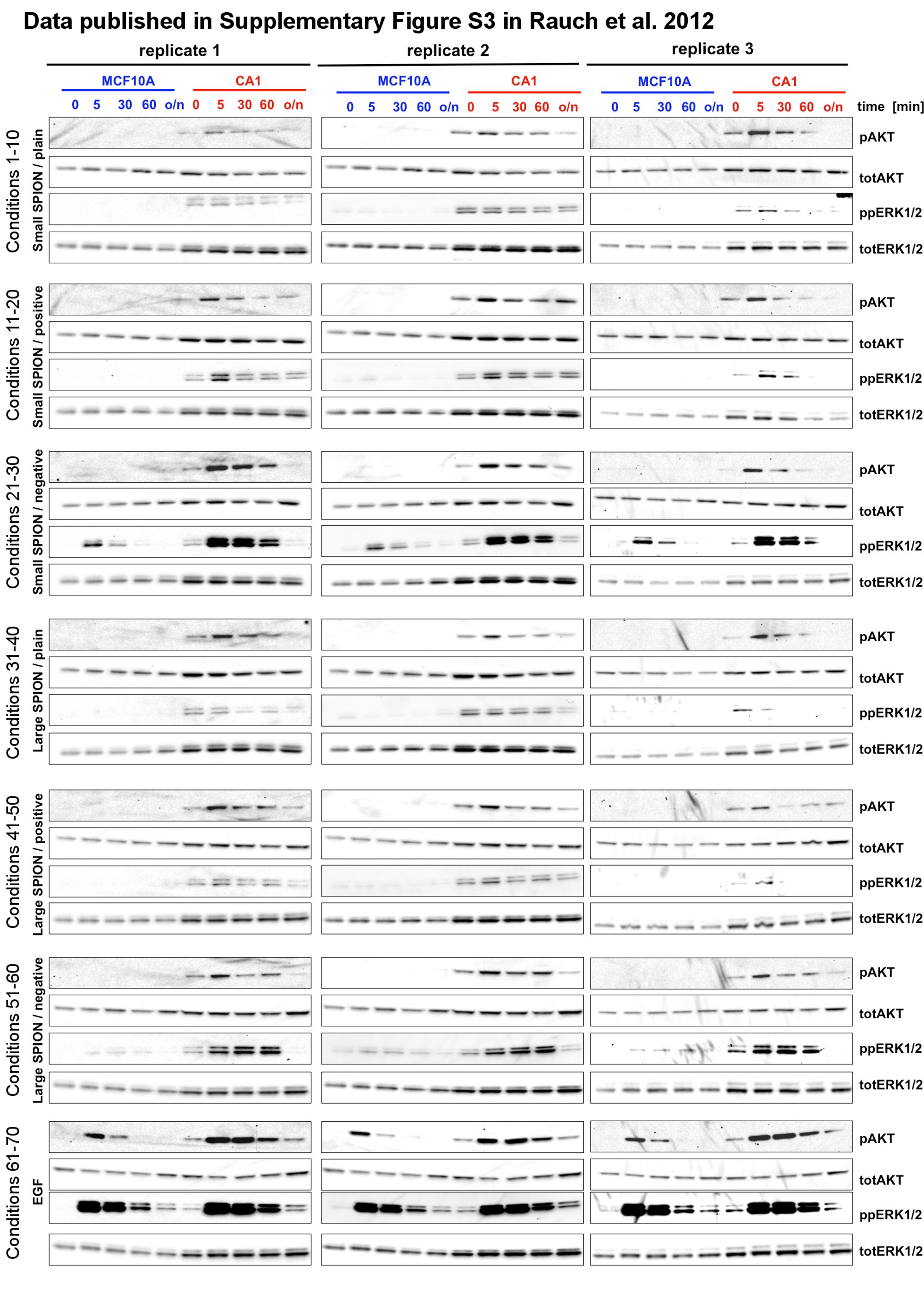

Supplement: Figure S7 — Figure S3 of [25] . Experimental data used in Figures 3C and 4. The experiments shown in Figure S5 were performed as described by Rauch et al. in [25]. (TIFF) [file pone.0087293.s007.tiff]

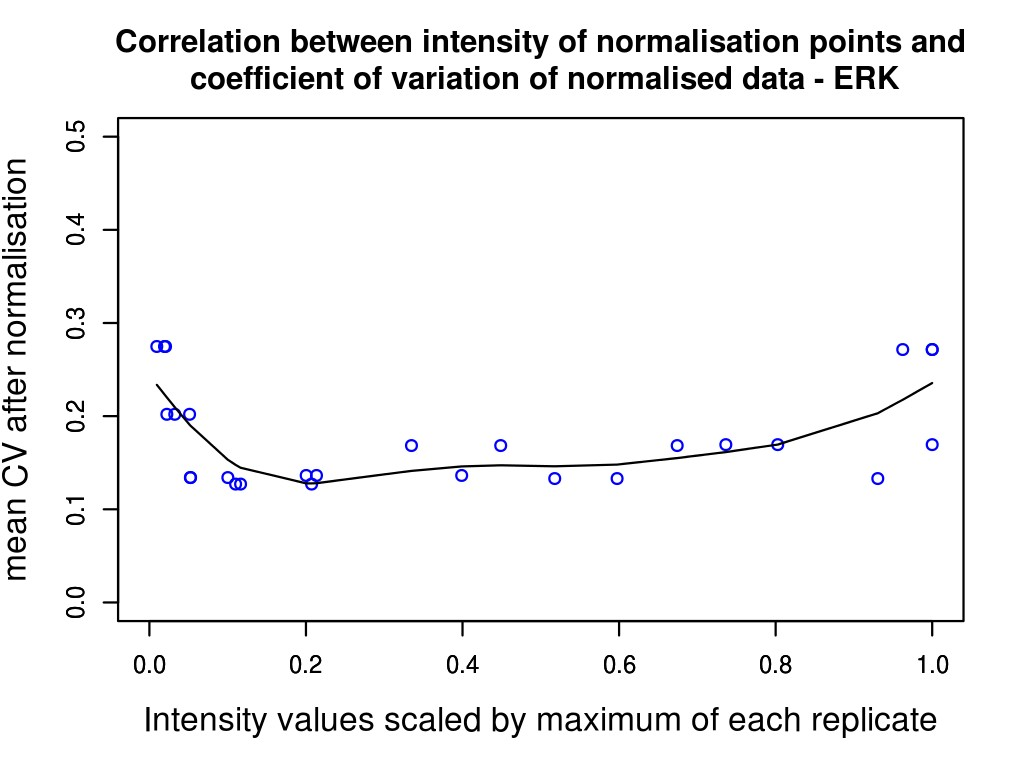

Supplement: Figure S8 — Correlation between the intensity of the normalisation points and the CV of the normalised data. Using data from the three replicates of the ERK dilution experiments detected with CCD imager, we tested every point on a blot as normalisation point. For each resulting normalisation we computed the average of the CV of the normalised data points, and plotted the value of each data point (scaled so that the maximum of each replicate is equal to 1) against the average CV obtained by normalising with the corresponding data point. The result shows how the intensities of each normalisation point chosen correlate with the variability of the normalised data. (TIFF) [file pone.0087293.s008.tiff]
