# Supplementary material for: Evaluating Strategies to Normalise Biological Replicates of Western Blot Data
Source: PLoS One. 2014 Jan 27;9(1):e87293. doi: 10.1371/journal.pone.0087293 (PMC3903630; doi:10.1371/journal.pone.0087293)
Supplement: Information S1 — Data and statistical analysis of dilution experiments for BSA, ERK, RSK1 and mTOR. (PDF) [file pone.0087293.s009.pdf]

Evaluating Strategies to Normalise Biological Replicates of Western Blot Data: Supporting Information S1

Andrea Degasperi[1,c], Marc R. Birtwistle[2], Natalia Volinsky[1], Jens Rauch[1], Walter Kolch[1,3,4] and Boris N. Kholodenko[1,3,4]

[1]Systems Biology Ireland, University College Dublin, Dublin, Republic of Ireland

[2] Department of Pharmacology and Systems Therapeutics, Icahn School of Medicine at Mount Sinai, New York, New York, United States of America

[3] Conway Institute of Biomolecular & Biomedical Research, University College Dublin, Dublin, Republic of Ireland

[4] School of Medicine and Medical Science, University College Dublin, Dublin, Republic of Ireland

[c] Corresponding author, e-mail address: andrea.degasperi@ucd.ie

Data and statistical analysis of dilution experiments for BSA, ERK, RSK1 and mTOR

SD standard deviation
CV coefficient of variation
S.E. standard error

		Raw optical densities			Data points tested for linearity	Regression lines of each replicate			Coefficients of determination			R^2 = 1 if data is linear			
**ECL**	dilution	Replicate 1	Replicate 2	Replicate 3		regression (1)	regression (2)	regression (3)	R^2 (1)	R^2 (2)	R^2 (3)	R^2 mean	R^2 SD	R^2 CV	R^2 S.E.
**CCD imager**	100	46777.668	64667.63	27749.22	full curve (10 samples)	y = 509.67x + 8403.8	y = 660.85x + 10818	y = 280.25x + 4200.1	0.7224	0.7973	0.843	0.78756667	0.06088631	0.07730941	0.03515273
	50	49021.96	58032.952	23294.078											
	25	38426.605	41051.333	16621.602	first 8 visible samples (7 dilutions, 128 fold)	y = 1500.4x + 1918.3	y = 1668.1x + 4305.6	y = 657.37x + 1776.7	0.975	0.9038	0.9325	0.9371	0.0358222	0.03822666	0.02068196
	12.5	20257.664	31330.12	11945.288	first 7 visible samples (6 dilutions, 64 fold)	y = 1678.8x + 1429.1	y = 2551.1x + 1884.6	y = 940.55x + 1000.3	0.9309	0.9595	0.9653	0.9519	0.0184163	0.01934688	0.01063265
	6.25	15424.744	21780.714	8128.146	first 6 visible samples (5 dilutions, 32 fold)	y = 2518.5x + 66.499	y = 3502.3x + 341	y = 1256.6x + 487.34	0.995	0.9946	0.9913	0.99363333	0.0020306	0.00204361	0.00117237
	3.125	8705.652	11839.915	4715.418											
	1.5625	4071.489	6661.459	2774.518											
	0.78125	2048.719	3172.518	1409.376											
	0.390625	836.263	1117.749	986.406											
	0.1953125	301.364	568.607	372.849											

SD standard deviation
CV coefficient of variation
S.E. standard error

		Raw optical densities			Data points tested for linearity	Regression lines of each replicate			Coefficients of determination			R^2 = 1 if data is linear			
**ECL**	dilution	Replicate 1	Replicate 2	Replicate 3		regression (1)	regression (2)	regression (3)	R^2 (1)	R^2 (2)	R^2 (3)	R^2 mean	R^2 SD	R^2 CV	R^2 S.E.
**Film**	100	76565.496	72547.253	86640.792	full curve (9/10 samples)	y = 647.21x + 24084	y = 606.55x + 24732	y = 813.31x + 23136	0.7383	0.6765	0.7119	0.7089	0.03100903	0.04374246	0.01790307
	50	69763.99	69398.505	83966.529											
	25	56074.413	54337.927	63660.032											
	12.5	42859.2	45855.957	50656.635	First 6 visible samples (5 dilutions, 32 fold)	y = 2808.7x + 12403	y = 3134.2x + 11708	y = 5822.4x + 6215.6	0.8119	0.8027	0.8763	0.8303	0.04010187	0.04829805	0.02315283
	6.25	35900.3	37056.229	38397.907	First 4 visible samples (3 dilutions, 8 fold)	y = 8029.7x + 4432.9	y = 9504x + 2194.4	y = 14064x - 250.31	0.9363	0.9459	0.9985	0.96023333	0.03348572	0.03487248	0.01933299
	3.125	28059.229	30329.35	30335.492	First 3 visible samples (2 dilutions, 4 fold)	y = 12589x + 766.54	y = 14369x - 1718.4	y = 14642x - 482.68	0.9863	0.983	0.9932	0.9875	0.00520481	0.00527069	0.003005
	1.5625	20102.087	20307.037	21632.057											
	0.78125	11603.702	10787.652	10820.116											
	0.390625	5015.731	3041.296	5645.853											
	0.1953125			2104.447											

SD standard deviation
CV coefficient of variation
S.E. standard error

		Raw optical densities			Data points tested for linearity	Regression lines of each replicate			Coefficients of determination			R^2 = 1 if data is linear			
**LiCOR**	dilution	Replicate 1	Replicate 2	Replicate 3		regression (1)	regression (2)	regression (3)	R^2 (1)	R^2 (2)	R^2 (3)	R^2 mean	R^2 SD	R^2 CV	R^2 S.E.
	100	64398.718	69060.697	62198.082	full curve (8/9 samples)	y = 653.03x + 6627	y = 675.31x + 8605.6	y = 618.84x + 7224	0.9115	0.9126	0.9142	0.91276667	0.00135769	0.00148745	0.00078387
	50	48644.262	50355.191	47313.262											
	25	31347.271	35981.806	30629.806											
	12.5	20985.179	22719.593	19069.179											
	6.25	13716.208	13050.551	12707.38	first 8 visible samples (7 dilutions, 128 fold)	y = 968.07x + 3645.8	y = 675.31x + 8605.6	y = 618.84x + 7224	0.9528	0.9126	0.9142	0.92653333	0.02276166	0.02456648	0.01314145
	3.125	6645.51	7326.167	5174.146	first 7 visible samples (6 dilutions, 64 fold)	y = 1271.4x + 1982.4	y = 990.47x + 5149.2	y = 926.62x + 3848.6	0.9515	0.9418	0.9629	0.95206667	0.01056141	0.01109314	0.00609763
	1.5625	2789.347	3445.832	2456.69	first 6 visible samples (5 dilutions, 32 fold)	y = 1742.7x + 452.88	y = 1410.8x + 2421.1	y = 1205.6x + 2037.6	0.9731	0.9779	0.9679	0.97296667	0.00500133	0.00514029	0.00288752
	0.78125	971.163	1438.477	1527.82											
	0.390625	496.678													
	0.1953125														

SD standard deviation
CV coefficient of variation
S.E. standard error

		Raw optical densities			Data points tested for linearity	Regression lines of each replicate			Coefficients of determination			R^2 = 1 if data is linear			
ECL	dilution	Replicate 1	Replicate 2	Replicate 3		regression (1)	regression (2)	regression (3)	R^2 (1)	R^2 (2)	R^2 (3)	R^2 mean	R^2 SD	R^2 CV	R^2 S.E.
CCD imager	100	15939.258	12679.744	14165.43	full curve (9 samples)	y = 161.26x + 2202.8	y = 125.59x + 3576.2	y = 141.63x + 1586.1	0.8426	0.5934	0.9117	0.78256667	0.16742677	0.2139457	0.09666389
	50	12793.945	13175.894	10430.945	first 8 visible samples (7 dilutions, 128 fold)	y = 261.22x + 1256.9	y = 270.14x + 2208.4	y = 207.87x + 959.26	0.8996	0.7963	0.9446	0.88016667	0.07603593	0.08638811	0.04389937
	25	9523.338	12260.066	7334.752	first 7 visible samples (6 dilutions, 64 fold)	y = 396.97x + 512.54	y = 496.37x + 967.88	y = 289.97x + 509.04	0.9452	0.9439	0.9739	0.95433333	0.01695769	0.01776915	0.00979053
	12.5	7153.217	8883.752	4742.56	first 6 visible samples (5 dilutions, 32 fold)	y = 574.8x - 64.605	y = 713.83x + 262.1	y = 372.44x + 241.39	0.9991	0.9905	0.9904	0.99333333	0.00499433	0.00502785	0.00288348
	6.25	3402.61	5256.267	2837.075											
	3.125	1856.761	2730.417	1560.104											
	1.5625	838.648	1320.447	741.598											
	0.78125	355.557	676.577	459.213											
	0.390625	151.071	272.142	273.314											
	0.1953125														

SD standard deviation
CV coefficient of variation
S.E. standard error

		Raw optical densities			Data points tested for linearity	Regression lines of each replicate			Coefficients of determination			R^2 = 1 if data is linear			
ECL	dilution	Replicate 1	Replicate 2	Replicate 3		regression (1)	regression (2)	regression (3)	R^2 (1)	R^2 (2)	R^2 (3)	R^2 mean	R^2 SD	R^2 CV	R^2 S.E.
Film	100	42141.492	36533.907	40090.614	full curve (6/7 samples)	y = 369.02x + 11663	y = 316.48x + 12460	y = 363.69x + 9780.9	0.6866	0.5896	0.7376	0.67126667	0.075182	0.1120002	0.04340635
	50	37274.714	36398.028	35483.291											
	25	32720.12	32228.563	28991.534	First 3 visible samples (2 dilutions, 4 fold)	y = 2129.3x - 5361.9	y = 2537.1x - 2597.6	y = 1713.6x - 3921	0.9969	0.9999	1	0.99893333	0.00176163	0.00176351	0.00101708
	12.5	21470.543	24903.614	17497.735	First 4 visible samples (3 dilutions, 8 fold)	y = 1412.6x - 750.62	y = 2138.6x - 1315.6	y = 1244x - 899.69	0.9514	0.9933	0.9731	0.9726	0.02095447	0.0215448	0.01209807
	6.25	7299.066	13231.572	6792.288	First 6 visible samples (5 dilutions, 32 fold)	y = 369.02x + 11663	y = 685.1x + 7674.5	y = 363.69x + 9780.9	0.6866	0.7727	0.7376	0.7323	0.04329399	0.05912057	0.0249958
	3.125	1723.861	5413.459	1431.719											
	1.5625		1311.426												
	0.78125														
	0.390625														
	0.1953125														

SD standard deviation
CV coefficient of variation
S.E. standard error

		Raw optical densities			Data points tested for linearity	Regression lines of each replicate			Coefficients of determination			R^2 = 1 if data is linear			
LiCOR	dilution	Replicate 1	Replicate 2	Replicate 3		regression (1)	regression (2)	regression (3)	R^2 (1)	R^2 (2)	R^2 (3)	R^2 mean	R^2 SD	R^2 CV	R^2 S.E.
	100	37868.668	43575.972	41042.923	full curve (8 samples, 7 dilutions, 128 fold)	y = 384.99x + 5032.3	y = 398.23x + 8258.6	y = 399.7x + 7273.8	0.8654	0.8816	0.8533	0.86676667	0.01419941	0.01638205	0.00819803
	50	31810.889	32572.709	35561.901	first 7 visible samples (6 dilutions, 64 fold)	y = 637.2x + 2266.3	y = 598.92x + 6057.6	y = 675.91x + 4244.6	0.9586	0.8991	0.9576	0.93843333	0.03406734	0.03630235	0.01966879
	25	21405.413	25775.546	24647.224	First 6 visible samples (5 dilutions, 32 fold)	y = 862.57x + 803.39	y = 932.9x + 3889.8	y = 917.12x + 2678.9	0.9823	0.9592	0.9815	0.97433333	0.01311195	0.01345736	0.00757019
	12.5	13153.543	18016.89	15543.191											
	6.25	7133.086	10425.099	9566.078											
	3.125	3620.631	7711.07	5943.007											
	1.5625	1527.761	5244.463	3581.865											
	0.78125	434.698	2081.66	1931.811											
	0.390625														
	0.1953125														

SD standard deviation
CV coefficient of variation
S.E. standard error

		Raw optical densities			Data points tested for linearity	Regression lines of each replicate			Coefficients of determination			R^2 = 1 if data is linear			
ECL	dilution	Replicate 1	Replicate 2	Replicate 3		regression (1)	regression (2)	regression (3)	R^2 (1)	R^2 (2)	R^2 (3)	R^2 mean	R^2 SD	R^2 CV	R^2 S.E.
CCD imager	100	7455.711	7866.104	7199.589	full curve (7 samples)	y = 77.74x + 536.06	y = 84.023x + 790.62	y = 74.419x + 457.63	0.9164	0.8437	0.9366	0.8989	0.048859902	0.054355214	0.028209277
	50	5697.175	7005.64	5203.518	6 samples (5 dilutions, 32 fold)	y = 119.32x - 3.7254	y = 148.6x - 47.73	y = 108.48x + 15.398	0.9828	0.9777	0.9842	0.981566667	0.003421013	0.003485258	0.001975123
	25	3457.569	4463.397	3205.74	5 samples (4 dilutions, 16 fold)	y = 148.74x - 235.68	y = 190.41x - 377.36	y = 134.68x - 191.18	0.9963	0.9955	0.9988	0.996866667	0.001721434	0.001726844	0.00099387
	12.5	1731.255	1877.376	1438.719											
	6.25	575.456	704.82	631.355											
	3.125	207.607	209.435	215.021											
	1.5625	54.121	80.95	76.95											
	0.78125														
	0.390625														
	0.1953125														

SD standard deviation
CV coefficient of variation
S.E. standard error

		Raw optical densities			Data points tested for linearity	Regression lines of each replicate			Coefficients of determination			R^2 = 1 if data is linear			
ECL	dilution	Replicate 1	Replicate 2	Replicate 3		regression (1)	regression (2)	regression (3)	R^2 (1)	R^2 (2)	R^2 (3)	R^2 mean	R^2 SD	R^2 CV	R^2 S.E.
Film	100	50217.099	44657.229	40941.673	6 samples (5 dilutions, 32 fold)	y = 418.37x + 16060	y = 339.32x + 16343	y = 296.86x + 16071	0.6923	0.7192	0.7254	0.7123	0.017595738	0.024702707	0.010158904
	50	46673.836	40040.844	36701.894											
	25	38217.593	34165.451	31259.238	First 4 visible samples (3 dilutions, 8 fold)	y = 1511.6x + 2746.2	y = 1188x + 6119.7	y = 1021.6x + 7334.3	0.9346	0.9308	0.922	0.929133333	0.006463229	0.006956191	0.003731547
	12.5	26873.643	24161.43	23420.359	First 3 visible samples (2 dilutions, 4 fold)	y = 2412.1x - 3047.5	y = 1830.7x + 1984.5	y = 1649x + 3297.5	0.9972	0.9562	0.9736	0.975666667	0.020577982	0.021091201	0.011880703
	6.25	12716.702	15547.752	15073.752											
	3.125	4030.933	6291.368	7470.782											
	1.5625														
	0.78125														
	0.390625														
	0.1953125														

SD standard deviation
CV coefficient of variation
S.E. standard error

		Raw optical densities			Data points tested for linearity	Regression lines of each replicate			Coefficients of determination			R^2 = 1 if data is linear			
**ECL**	dilution	Replicate 1	Replicate 2	Replicate 3		regression (1)	regression (2)	regression (3)	R^2 (1)	R^2 (2)	R^2 (3)	R^2 mean	R^2 SD	R^2 CV	R^2 S.E.
**CCD imager**	100	5766.933	6245.175	5377.569	full curve (7 samples)	y = 63.733x + 307.64	y = 64.037x + 1292.5	y = 55.061x + 821.98	0.8766	0.6663	0.8091	0.784	0.10737332	0.13695577	0.06199202
	50	5174.104	6197.468	5090.69	6 samples (5 dilutions, 32 fold)	y = 108.22x - 269.87	y = 134.66x + 375.69	y = 101.32x + 221.42	0.9983	0.8664	0.9574	0.9407	0.06751718	0.07177334	0.03898106
	25	2416.669	5456.933	2842.376	5 samples (4 dilutions, 16 fold)	y = 104.55x - 240.95	y = 235.79x - 421.69	y = 123.17x + 49.172	0.993	0.9955	0.907	0.96516667	0.05038932	0.05220789	0.02909229
	12.5	1018.355	2662.79	2241.962											
	6.25	302.092	811.406	782.82											
	3.125	92.192	316.92	259.385											
	1.5625	30.121	64.364	85.192											
	0.78125														
	0.390625														
	0.1953125														

SD standard deviation
CV coefficient of variation
S.E. standard error

		Raw optical densities			Data points tested for linearity	Regression lines of each replicate			Coefficients of determination			R^2 = 1 if data is linear			
**ECL**	dilution	Replicate 1	Replicate 2	Replicate 3		regression (1)	regression (2)	regression (3)	R^2 (1)	R^2 (2)	R^2 (3)	R^2 mean	R^2 SD	R^2 CV	R^2 S.E.
**Film**	100	19216.551	27271.108	27337.673	First 6 visible samples (5 dilutions, 32 fold)	y = 188.29x + 3812.2	y = 533.74x + 4469.4	y = 262.31x + 5518.6	0.7473	0.7975	0.7576	0.76746667	0.02651459	0.03454819	0.0153082
	50	18756.137	27083.037	24934.309	First 5 visible samples (4 dilutions, 16 fold)	y = 378.29x + 815.9	y = 985.76x + 905.16	y = 507.09x + 1658.3	0.9293	0.9214	0.9015	0.9174	0.01432515	0.01561495	0.00827063
	25	11253.288	23475.622	17494.51	First 4 visible samples (3 dilutions, 8 fold)	y = 511.1x - 496.95	y = 1551.2x - 1889.5	y = 790.49x - 1143.2	0.8835	0.9867	0.9317	0.93396667	0.05163733	0.05528819	0.02981282
	12.5	8581.267	17013.359	11809.146	First 3 visible samples (2 dilutions, 4 fold)	y = 910.21x - 3064.8	y = 1929.6x - 3106.7	y = 1227.5x - 3954.7	0.974	0.9921	0.9652	0.9771	0.01371532	0.01403676	0.00791854
	6.25	1818.79	9108.167	2455.912											
	3.125	316.435	2457.811	721.941											
	1.5625		218.435												
	0.78125														
	0.390625														
	0.1953125														
