# Supplementary material for: Evaluating Strategies to Normalise Biological Replicates of Western Blot Data
Source: PLoS One. 2014 Jan 27;9(1):e87293. doi: 10.1371/journal.pone.0087293 (PMC3903630; doi:10.1371/journal.pone.0087293)
Supplement: Information S2 — Mathematical Supplement. This document contains a characterisation of the normalisation by sum as an optimisation problem, an analytical solution of the normalisation by least squared difference optimisation and a description of how the results in Figures S5 and S6 were obtained using normal distributions. (DOC) [file pone.0087293.s010.doc]

**Evaluating Strategies to Normalise Biological Replicates of Western Blot Data: Supporting Information S2**

Andrea Degasperi^1,c^, Marc R. Birtwistle^2^, Natalia Volinsky^1^, Jens Rauch^1^, Walter Kolch^1,3,4^ and Boris N. Kholodenko^1,3,4^

^1^Systems Biology Ireland, University College Dublin, Dublin, Republic of Ireland

^2^Department of Pharmacology and Systems Therapeutics, Icahn School of Medicine at Mount Sinai, New York, New York, United States of America

^3^ Conway Institute of Biomolecular & Biomedical Research, University College Dublin, Dublin, Republic of Ireland

^4^ School of Medicine and Medical Science, University College Dublin, Dublin, Republic of Ireland

^c^ Corresponding author, e-mail address: andrea.degasperi@ucd.ie

**Section S1. Normalisation by Sum of the Replicate as an Optimisation Problem**

Recall that $i\in I$is the index for the experiments on the same blot, while $j\in J$is the index for the replicates of the same blot. We know that the replicates are samples from different distributions: $d_{i}^{j}$ is a sample from the random variables $D_{i}^{j}$, with $D_{i}^{j}=\alpha_{j}\cdot\left[S\right]_{i}$. Assuming we have $\left|J\right|$ replicates, a formalisation of the normalisation by sum in terms of an objective function to be minimised is:

$obj_{1}\left(\beta_{2},...,\beta_{\left|J\right|}\right)=\sum_{j\in J\backslash\left\{1\right\}} \left(\sum_{i\in I} \beta_{j}\cdot d_{i}^{j}-\sum_{i\in I} \beta_{1}\cdot d_{i}^{1}\right)^{2}$, with $\beta_{1}=\frac{1}{\sum_{i\in I} d_{i}^{1}}$

Because $\beta_{1}$ is set to $1/\sum_{i\in I} d_{i}^{1}$, the above objective function implies that we are normalising the data in replicate 1 by sum and that we want to scale the other replicates by factors $\beta_{2},...,\beta_{\left|J\right|}$ such that the difference between the sum of each replicate and the sum of the first replicate is minimised. Notice that it is necessary to set $\beta_{1}$ to a constant value to avoid the trivial solution $\beta_{j}=0$for all $j\in J$.

We minimise $obj_{1}$ to find the optimal $\beta_{2},...,\beta_{\left|J\right|}$. The result of this optimisation can be computed analytically and yields $\beta_{j}=\frac{1}{\sum_{i\in I} d_{i}^{j}}$for all $j\in J$.

PROOF: We assume that the $\beta_{2},...,\beta_{\left|J\right|}$ are constants, which implies that their derivative with respect to other variables is zero. Thus, assuming$\forall j,k\in J,j\neq k.\frac{d}{d\beta_{j}}\beta_{k}=0$, then for all $j\in J\backslash\{1\}$:

$$\frac{d}{d\beta_{j}}obj_{1}=2\cdot\left(\sum_{i\in I} \beta_{j}\cdot d_{i}^{j}-\sum_{i\in I} \beta_{1}\cdot d_{i}^{1}\right)\cdot\left(\sum_{i\in I} d_{i}^{j}\right)=0$$

$$0=\beta_{j}\cdot\left(\sum_{i\in I} d_{i}^{j}\right)-\beta_{1}\cdot\left(\sum_{i\in I} d_{i}^{1}\right)$$

| $\beta_{j}=\frac{\beta_{1}\cdot\left(\sum_{i\in I} d_{i}^{1}\right)}{\left(\sum_{i\in I} d_{i}^{j}\right)}=\frac{1}{\sum_{i\in I} d_{i}^{j}}$ | $\forall j\in J$ | (S1) |
| --- | --- | --- |

□

For all $j\in J$, $\beta_{j}$ is a sample from the random variable $\frac{1}{\sum_{i\in I} \alpha_{j}\cdot\left[S\right]_{i}}$. After normalisation, we obtain the normalised data $\tilde{d}_{i}^{j}=\beta_{j}\cdot d_{i}^{j}$, which are samples from the following random variables:

| $\tilde{D}_{i}=\beta_{j}\cdot\alpha_{j}\cdot\left[S\right]_{i}=\frac{\alpha_{j}\cdot\left[S\right]_{i}}{\sum_{k\in I} \alpha_{j}\cdot\left[S\right]_{k}}=\frac{\left[S\right]_{i}}{\sum_{k\in I} \left[S\right]_{k}}$ | $\forall i\in I$ | (S2) |
| --- | --- | --- |

In this case the normalised data are comparable across blot replicates since the normalized random variables do not depend on the index *j*.

# Section S2. Normalisation by Least Squared Difference Optimisation

Recall that $i\in I$is the index for the experiments on the same blot, while $j\in J$is the index for the replicates of the same blot. Assuming we have $\left|J\right|$ replicates and we want to align every replicate to the first replicate, the objective function is as follows:

$obj_{2}\left(\beta_{2},...,\beta_{\left|J\right|}\right)=\sum_{j\in J\backslash\left\{1\right\}} \sum_{i\in I} \left(\beta_{j}\cdot d_{i}^{j}-\beta_{1}\cdot d_{i}^{1}\right)^{2}$, with $\beta_{1}=1$

We minimise $obj_{2}$ to find the optimal $\beta_{2},...,\beta_{\left|J\right|}$. The result of this optimisation can be computed analytically and yields $\beta_{j}=\frac{\sum_{i\in I} (d_{i}^{1}\cdot d_{i}^{j})}{\sum_{i\in I} (d_{i}^{j})^{2}}$for all $j\in J\backslash\{1\}$.

PROOF: We assume that the $\beta_{2},...,\beta_{\left|J\right|}$ are constants, which implies that their derivative with respect to other variables is zero. Thus, assuming $\forall j,k\in J,j\neq k.\frac{d}{d\beta_{j}}\beta_{k}=0$, then for all $j\in J\backslash\{1\}$:

$$\frac{d}{d\beta_{j}}obj_{2}=2\cdot\sum_{i\in I} \left(\beta_{j}\cdot d_{i}^{j}-\beta_{1}\cdot d_{i}^{1}\right)\cdot\left(d_{i}^{j}\right)=0$$

$$0=\beta_{j}\cdot\left(\sum_{i\in I} (d_{i}^{j})^{2}\right)-\beta_{1}\cdot\left(\sum_{i\in I} (d_{i}^{1}\cdot d_{i}^{j})\right)$$

| $\beta_{j}=\frac{\beta_{1}\cdot\left(\sum_{i\in I} (d_{i}^{1}\cdot d_{i}^{j})\right)}{\left(\sum_{i\in I} (d_{i}^{j})^{2}\right)}=\frac{\sum_{i\in I} (d_{i}^{1}\cdot d_{i}^{j})}{\sum_{i\in I} (d_{i}^{j})^{2}}$ | $j\in J\backslash\{1\}$ | (S3) |
| --- | --- | --- |

□

For all $j\in J\backslash\{1\}$, $\beta_{j}$ is a sample from the random variable $\frac{\sum_{i\in I} (\alpha_{1}\cdot[S]_{i,1}\cdot\alpha_{j}\cdot[S]_{i,j})}{\sum_{i\in I} (\alpha_{j}\cdot[S]_{i,j})^{2}}$, where $[S]_{i,1}$and $[S]_{i,j}$are independent random variables distributed as $\left[S\right]_{i}$. Random variables $[S]_{i,1}$and $[S]_{i,j}$are independent because we have in the same expression data from two replicates, which we consider independent samples of $\left[S\right]_{i}$. Additionally, different $\beta_{j}$ will be a function of the same $[S]_{i,1}$, making them dependent on each other.

We can also observe that:

$$\beta_{1}=1=\frac{\sum_{i\in I} (d_{i}^{1}\cdot d_{i}^{1})}{\sum_{i\in I} (d_{i}^{1})^{2}}$$

Which implies $\beta_{1}$is a sample from the random variable $\frac{\sum_{i\in I} (\alpha_{1}\cdot[S]_{i,1}\cdot\alpha_{1}\cdot[S]_{i,1})}{\sum_{i\in I} (\alpha_{1}\cdot[S]_{i,1})^{2}}$.

Thus, we can write that for all $j\in J$:

| $\beta_{j}=\frac{\sum_{i\in I} (d_{i}^{1}\cdot d_{i}^{j})}{\sum_{i\in I} (d_{i}^{j})^{2}}$ | $\forall j\in J$ | (S4) |
| --- | --- | --- |

We know that the replicates are samples from different random variables: $d_{i}^{j}$ is a sample from distribution $D_{i}^{j}$, with $D_{i}^{j}=\alpha_{j}\cdot\left[S\right]_{i}$. After normalisation, we obtain the normalised data $\tilde{d}_{i}^{j}=\beta_{j}\cdot d_{i}^{j}$, which are samples from the random variables$\tilde{D}_{i}^{j}=\beta_{j}\cdot\alpha_{j}\cdot\left[S\right]_{i}$ ($\beta_{1}=1$). While $\tilde{D}_{i}^{1}=D_{i}^{1}$, for all $j\in J\backslash\left\{1\right\}$,

$$\tilde{D}_{i}^{j}=\frac{\sum_{i\in I} (\alpha_{1}\cdot[S]_{i,1}\cdot\alpha_{j}\cdot[S]_{i,j})}{\sum_{i\in I} (\alpha_{j}\cdot[S]_{i,j})^{2}}\cdot\alpha_{j}\cdot[S]_{i,j}$$

$$=\alpha_{j}\cdot[S]_{i,j}\cdot\frac{\alpha_{1}\cdot\alpha_{j}\cdot\sum_{i\in I} ([S]_{i,1}\cdot[S]_{i,j})}{\alpha_{j}^{2}\cdot\sum_{i\in I} ([S]_{i,j})^{2}}$$

| $=\alpha_{1}\cdot[S]_{i,j}\cdot\frac{\sum_{i\in I} ([S]_{i,1}\cdot[S]_{i,j})}{\sum_{i\in I} ([S]_{i,j})^{2}}$ | $\forall i\in I$ | (S5) |
| --- | --- | --- |

where $[S]_{i,1}$and $[S]_{i,j}$are independent random variables distributed as $\left[S\right]_{i}$. Notice that the normalised data is all proportional to $\left[S\right]_{i}$ in the same way, that is with constant of proportionality $\alpha_{1}$.

**Section S3. Use of normal distributions in place of log-normal distributions in the simulated scenarios**

In this section we use a simulated scenario where the responses to eight treatments have the same mean and variance as in Figure 3A of the main text. The distribution used here is normal instead of log-normal. If random variable$Y$ is normally distributed, then $R=X/Y$has undefined mean and variance because $Y$ can assume values arbitrarily close to zero. In practice, we use a normal distribution truncated at a point which is 1% of the coefficient of variation of $X$ or $Y$. It has been shown that for coefficient of variations up to 0.3, the difference between truncated and non-truncated normal distribution is negligible .

We reproduce the equivalent of Figures 3B,D and 5 of the main text using normal distributions. The results are shown in Figures S5A,B and S6.

**References**

1. Geary RC (1930) The frequency distribution of the quotient of two normal variates. Journal of the Royal Statistical Society 93: 442-446.

2. Hall RL (1979) Inverse moments for a class of truncated normal distributions. Sankhya: The Indian Journal of Statistics 41: 66-76.
