# Supplementary material for: Evaluating Strategies to Normalise Biological Replicates of Western Blot Data
Source: PLoS One. 2014 Jan 27;9(1):e87293. doi: 10.1371/journal.pone.0087293 (PMC3903630; doi:10.1371/journal.pone.0087293)
Supplement: Information S3 — Guide lines for the quantification of Western blots. (DOC) [file pone.0087293.s011.doc]

**Evaluating Strategies to Normalise Biological Replicates of Western Blot Data: Supporting Information S3**

Andrea Degasperi^1,c^, Marc R. Birtwistle^2^, Natalia Volinsky^1^, Jens Rauch^1^, Walter Kolch^1,3,4^ and Boris N. Kholodenko^1,3,4^

^1^Systems Biology Ireland, University College Dublin, Dublin, Republic of Ireland

^2^Department of Pharmacology and Systems Therapeutics, Icahn School of Medicine at Mount Sinai, New York, New York, United States of America

^3^ Conway Institute of Biomolecular & Biomedical Research, University College Dublin, Dublin, Republic of Ireland

^4^ School of Medicine and Medical Science, University College Dublin, Dublin, Republic of Ireland

^c^ Corresponding author, e-mail address: [andrea.degasperi@ucd.ie](mailto:andrea.degasperi@ucd.ie)

**Guide lines for the Quantification of Western Blots**

1. Separate samples by SDS-PAGE and transfer to membrane.
2. Use primary, secondary antibodies and ECL kits (or fluorescent secondary antibodies) according to manufacturer specifications.
3. Detect bands using either X-ray film, CCD imager or fluorescence scanner (for fluorescent secondary antibodies) and quantify by densitometry with imaging software such as ImageJ. Consider the following guidelines.
4. X-ray film for quantitative Western blotting should be limited to the case in which the intensities vary experimentally not more than 4 to 8 fold. This is because it is difficult to prevent overexposure that is due to saturated film.
5. CCD imager has a wider linear range than X-ray film. The automatic image acquisition system collects a series of pictures of different exposure length and detects when high intensity bands reach saturation. Thus, overexposure can be avoided using only the pictures that are free from saturated bands.
6. If using ECL with CCD imager, and the dynamic range of the collected data is more than 32-fold (see Results in Main Text), then the data are likely to exceed the linear range. This is due to the ECL reaction alone and the use of fluorescent secondary antibodies should be considered. Alternatively, one can design a dilution experiment to determine whether in the conditions considered the dynamic range of the protein of interest is linear.
7. Divide data points by corresponding loading control to correct for minor differences in cell numbers loaded in the gels.
8. Normalise replicates of membranes in order to compare them. Consider the following guidelines.
9. **Normalisation by fixed point or control.** Divide all data points from a replicate by a normalisation point in that replicate. The normalisation point can be the untreated or neutral condition that should be present on every blot. Any other condition is also fine as long as it is present on every blot and it is one of the most reproducible conditions. The variance of the normalised data depends on the choice of the normalisation point, so it is good practice to try different normalisation points. Low intensity data points should be avoided as normalisation points as they usually lead to high variance in the normalised data, because they tend to have a low signal-to-noise ratio. If there is a risk of non-linearity (points 3a and 3c above), it is better to avoid high intensity data points. In this case, medium intensity points should be preferred.
10. **Normalisation by sum of the replicate.** Divide all data points from a replicate by the sum of all data points in that replicate. This normalisation usually leads to low variance of the normalised data. It should be noted that the variance of high intensity measurements will be underestimated. This will affect hypothesis testing, increasing the false positives in the t-test when testing high intensity measurements. In this particular case, a p-value of 0.05 might be not enough to deduce a statistically significant difference between samples.
11. **Normalisation by optimal alignment.** Compute a scaling factor for each replicate by optimising a function that describes an alignment of the replicates. For example, find the scaling factors that minimise the squared differences between replicates or that minimise the coefficient of variation of the normalised data. This type of normalisation presents the same properties, advantages and disadvantages of the normalisation by sum of the replicate. Thus, the use of normalisation by sum is advised, just because it is much simpler to apply.
